# Caveolin‐3 loss linked with the P104L LGMD‐1C mutation modulates skeletal muscle mTORC1 signalling and cholesterol homeostasis

**DOI:** 10.1002/jcsm.13317

**Published:** 2023-09-06

**Authors:** Dinesh S. Shah, Raid B. Nisr, Gabriela Krasteva‐Christ, Harinder S. Hundal

**Affiliations:** ^1^ Division of Cell Signalling and Immunology, Sir James Black Centre, School of Life Sciences University of Dundee Dundee DD1 5EH UK; ^2^ Institute of Anatomy and Cell Biology, School of Medicine Saarland University Homburg Germany

**Keywords:** amino acid, caveolin‐3, caveolinopathy, LGMD‐1C, lysosome, mTORC1, skeletal muscle

## Abstract

**Background:**

Caveolins are the principal structural components of plasma membrane caveolae. Dominant pathogenic mutations in the muscle‐specific caveolin‐3 (Cav3) gene isoform, such as the limb girdle muscular dystrophy type 1C (LGMD‐1C) P104L mutation, result in dramatic loss of the Cav3 protein and pathophysiological muscle weakness/wasting. We hypothesize that such muscle degeneration may be linked to disturbances in signalling events that impact protein turnover. Herein, we report studies assessing the effects of Cav3 deficiency on mammalian or mechanistic target of rapamycin complex 1 (mTORC1) signalling in skeletal muscle cells.

**Methods:**

L6 myoblasts were stably transfected with Cav3^P104L^ or expression of native Cav3 was abolished by CRISPR/Cas9 genome editing (Cav3 knockout [Cav3KO]) prior to performing subcellular fractionation and immunoblotting, analysis of real‐time mitochondrial respiration or fixed cell immunocytochemistry. Skeletal muscle from wild‐type and Cav3^−/−^ mice was processed for immunoblot analysis of downstream mTORC1 substrate phosphorylation.

**Results:**

Cav3 was detected in lysosomal‐enriched membranes isolated from L6 myoblasts and observed by confocal microscopy to co‐localize with lysosomal‐specific markers. Cav3^P104L^ expression, which results in significant (~95%) loss of native Cav3, or CRISPR/Cas9‐mediated Cav3KO, reduced amino acid‐dependent mTORC1 activation. The decline in mTORC1‐directed signalling was detected by immunoblot analysis of L6 muscle cells and gastrocnemius Cav3^−/−^ mouse muscle as judged by reduced phosphorylation of mTORC1 substrates that play key roles in the initiation of protein synthesis (4EBP1^S65^ and S6K1^T389^). S6K1^T389^ and 4EBP1^S65^ phosphorylation reduced by over 75% and 80% in Cav3KO muscle cells and by over 90% and 30% in Cav3^−/−^ mouse skeletal muscle, respectively. The reduction in protein synthetic capacity in L6 muscle cells was confirmed by analysis of puromycylated peptides using the SUnSET assay. Cav3 loss was also associated with a 26% increase in lysosomal cholesterol, and pharmacological manipulation of lysosomal cholesterol was effective in replicating the reduction in mTORC1 activity observed in Cav3KO cells. Notably, re‐expression of Cav3 in Cav3KO myoblasts normalized lysosomal cholesterol content, which coincided with a recovery in protein translation and an associated increase in mTORC1‐directed phosphorylation of downstream targets.

**Conclusions:**

Our findings indicate that Cav3 can localize on lysosomal membranes and is a novel regulator of mTORC1 signalling in muscle. Cav3 deficiency associated with the Cav3^P104L^ mutation impairs mTORC1 activation and protein synthetic capacity in skeletal muscle cells, which may be linked to disturbances in lysosomal cholesterol trafficking and contribute to the pathology of LGMD‐1C.

## Introduction

Caveolin‐3 (Cav3) is regarded as a crucial component of muscle physiology as underscored by mutations within the *CAV3* gene that result in numerous pathologies, commonly referred to as caveolinopathies. Mutations causing a loss of functional Cav3 can result in rippling muscle disease, familial hypertrophic cardiomyopathy, distal myopathy, isolated hyperCKaemia and limb girdle muscular dystrophy type 1C (LGMD‐1C).^1^ In LGMD‐1C, a single missense amino acid (AA) substitution from a proline to a leucine at position 104 (Cav3^P104L^), which is inherited in an autosomal dominant manner, leads to myopathic changes that contribute to atrophy and weakness of muscle.^2^ However, little is known regarding the mechanism by which the Cav3^P104L^ mutation contributes to muscle atrophy.

Whilst caveolin (Cav) proteins are classically thought to be associated with the plasma membrane, there is increasing appreciation that they can also localize to numerous subcellular membrane compartments including, for example, the Golgi,^3^ endosomes,^4^ cytosolic lipid droplets,^5^ mitochondrial associated membranes^6^, ^7^ and lysosomes.^8^ The lysosome represents a membrane‐bound dynamic organelle that is primarily recognized as a vital component in the degradative process known as autophagy, which acts to degrade and recycle damaged components of the cell including organelles and proteins to generate a reusable pool of AAs.^9^ Autophagy can be activated upon nutrient/energy starvation but has also been recognized as essential for innate immunity and cellular growth.^9^, ^10^ Lysosomes facilitate autophagy by exposing intracellular components to an acidic luminal environment (pH 4.5–5.0), which is sustained by a vacuolar (v)‐ATPase that pumps protons into the lysosomal lumen.^11^ In addition to being involved in recycling cellular components, lysosomes are also involved in processing extracellular low‐density lipoproteins (LDLs) following receptor‐mediated endocytosis and thereby contribute to cellular cholesterol homeostasis.^12^ Significantly, lysosomal pH and cholesterol content have been identified as being important in influencing lysosomal association of caveolin‐1 (Cav1). In Chinese hamster ovary (CHO) cells, Cav1 accumulates at the surface of late endosomes or lysosomes when cells are deprived of serum.^8^ Removal of serum induces an elevation in lysosomal pH, a factor considered partly responsible for the redistribution of Cav1 to lysosomes as the latter is also initiated in response to lysosomal pH raising drugs (i.e., v‐ATPase inhibitor bafilomycin A_1_). It is unlikely that this pH‐regulated lysosomal association of Cav1 is intended to promote Cav1 degradation given that serum repletion induces Cav1 dissociation from the lysosomal compartment.^8^ Accumulation of Cav1 at lysosomes can also be promoted in cells in which the egress of lysosomal cholesterol is inhibited or under circumstances when cellular cholesterol is pharmacologically depleted using methyl‐β‐cyclodextrin (MBC).^8^ This latter observation may be a consequence of the fact that Cav proteins possess a cholesterol binding domain and have been implicated in the maintenance of membrane cholesterol,^5^, ^13^ a process that may itself rely upon Cav proteins shuttling cholesterol between subcellular membrane compartments, such as the lysosome and plasma membrane.^8^

The association of Cav1 with lysosomes may also influence the process of autophagy. A critical final step in the autophagic process involves the fusion of autophagosomes with lysosomes, and several studies have implicated Cav1 as an important modulator of the autophagic event.^14^, ^15^ Whilst it is currently unknown whether Cav3 similarly associates with lysosomes in skeletal muscle cells, there is evidence that it protects against cardiac ischaemia via modulation of autophagy markers beclin‐1, Atg12–Atg5 and light chain (LC3‐II).^16^ Notably, over‐expression of Cav3 increases the prevalence of autophagy markers whilst Cav3 silencing results in suppression of autophagy and increased expression of apoptotic proteins by, as yet, poorly understood mechanisms.^16^

The lysosome has also emerged as a critical site for the activation of the mammalian or mechanistic target of rapamycin complex 1 (mTORC1), which integrates numerous environmental (i.e., nutritional, hormonal and stress) cues to promote anabolic processes that facilitate cell/tissue growth (e.g., protein synthesis) or suppress catabolic processes, such as lysosomal biogenesis and autophagy.^17^ Significantly, the importance of lysosomal cholesterol in the activation of the mTORC1 complex has also been reported.^18^ As Cav1 can interact with the lysosomal membrane in a regulated manner and may modulate lysosomal cholesterol trafficking and autophagy, we postulated that effective mTORC1 activation may also be critically dependent upon the lysosomal association of Cav proteins.^14^, ^16^ In the current study, we have tested this proposition by investigating whether, like Cav1, Cav3 also interacts with the lysosomal compartment and, if so, whether loss/dysfunction of Cav3 in skeletal muscle cells impacts upon mTORC1‐directed signalling and key regulatory endpoints such as protein synthesis and cell growth.

## Materials and methods

### Chemicals and reagents

Culture media (α‐MEM [α‐minimal essential medium]) the precise nutrient formulation of which can be sourced from the manufacturer (Cat: #11900073), OPTI‐MEM, foetal bovine serum (FBS) and antibiotic solutions were purchased from Life Technologies (Paisley, UK). Earle's balanced salt solution (EBSS) was obtained from Sigma‐Aldrich. [^3^H]‐leucine and [^14^C]‐α‐methyl‐aminoisobutyric acid (Me‐AIB) were purchased from PerkinElmer‐NEN (Bucks, UK). Primer oligonucleotides were synthesized by the oligonucleotide synthesis unit at the University of Dundee. Restriction enzymes, DNA polymerases and T4 DNA ligases were purchased from New England Biolabs.

### Animals

Cav3 knockout (Cav3KO) mice were produced by targeting exon‐2 of the Cav3 gene using the gene targeting approach detailed previously.^19^ Mice were maintained according to the German guidelines for the care and use of laboratory animals and experiments approved by the Saarland's Institutional Animal Care and Use Committee. Mice were anaesthetized using isoflurane and killed by severing the aorta within the thoracic cavity. Gastrocnemius muscle was removed from hindlimbs and immediately frozen in liquid nitrogen and stored at −80°C until necessary.

### Muscle cell culture

L6 muscle cells were cultured in α‐MEM containing 2% (v/v) FBS and 1% (v/v) antibiotic/antimycotic solution (100 units/mL of penicillin, 100 μg/mL of streptomycin and 250 ng/mL of amphotericin B) at 37°C with 5% CO_2_. Myoblasts were typically used 4 days post‐seeding. U18666a was added dropwise to tissue culture medium to a final concentration of 2.5 μg/mL and incubated with myoblasts overnight for 16 h. Cells were depleted of cellular cholesterol using 5 mM of MBC for 2 h prior to AA or serum withdrawal.

### Generation of Hemagglutinin (HA)‐tagged Transmembrane protein 192 (TMEM192) and cellular tools

Wild‐type (WT) myoblasts were transfected with the pBABE hygro 3xHA GFP vector (University of Dundee, MRC PPU Reagents and Services [DU55543]) into which the rat TMEM192 was cloned using BamHI and NotI restriction sites. Myoblasts were transfected with this construct using lipofectamine 3000 and selected for using 500 μg/mL of hygromycin and maintained in 50 μg/mL thereafter. Cav3KO muscle cells and those either stably expressing the Cav3^P104L^ mutation or empty vector (EV) were generated as previously described.^7^

### Sodium dodecyl sulfate‐polyacrylamide gel electrophoresis (SDS‐PAGE) and immunoblotting

Muscle cells were incubated and treated as described in the text and figure legends. After treatments, muscle cells were washed twice with ice‐cold phosphate‐buffered saline (PBS) and then lysed as described previously or used for preparation of total cell membranes.^7^ Protein concentration in cell lysates and membrane preparations was determined using the Bradford method.^20^ Cell lysates and membrane preparations were subject to SDS‐PAGE and separated proteins transferred onto polyvinylidene difluoride (PVDF) membrane (Millipore) prior to immunoblotting with the primary antibodies, diluted at 1:1000 in wash buffer containing 5% bovine serum albumin (BSA) (unless stated otherwise), as follows: Actin (#A5060) (diluted at 1:2000) was obtained from Sigma; GAPDH (#SC32233) (diluted at 1:2000) was purchased from Santa Cruz; TOM20 (#42406S), HA (#2367S), BiP (#C50B12), RCAS1 (#12290), phospho P70 S6K Thr 389 (#9205), P70 S6K1 (#9202), Phospho‐ULK1 Ser757 (#14202), ULK1 (#8054), Phospho‐4E‐BP1 Ser65 (#9451), 4E‐BP1 (#9452) and mTOR (#2983) were purchased from Cell Signaling Technology; Cav1 (#610058) and Cav3 (#610421) were purchased from BD Biosciences. LAMP1 (lysosomal‐associated membrane protein 1) (#ab24170) was purchased from Abcam. The α‐subunit of the Na,K‐ATPase antibody was from DSHB (University of Iowa). Puromycin (#MABE343) was purchased from Merck Millipore. Primary antibody detection was performed using appropriate horseradish peroxidase (HRP)‐conjugated secondary mouse (#7076S) or rabbit (#7074S) antibodies purchased from Cell Signaling Technology and visualized using enhanced chemiluminescence (Pierce‐Perbio Biotech, Tattenhall, UK) by exposure to autoradiographic film (Konica Minolta, Tokyo, Japan) or LICOR detection. Quantification of immunoblots was carried out using ImageJ (NIH).

### Analysis of cellular protein synthesis

Protein synthesis was determined by assaying puromycin incorporation into newly synthesized peptides.^21^ L6 myoblasts were pre‐incubated in the absence or presence of cycloheximide (50 μg/mL) prior to incubation with or without 1 μM of puromycin for 15 min. Following this incubation period, muscle cells were lysed and lysates subject to SDS‐PAGE and immunoblotting using a monoclonal anti‐puromycin antibody.

### Fixed cell imaging

Myoblasts were grown to 70% confluence in cell culture dishes on 13‐mm coverslips prior to being fixed using 4% (w/v) paraformaldehyde in PBS at room temperature (RT) for 15 min. Following fixation, myoblasts were permeabilized in PBS containing 0.1% (v/v) Triton for 15 min with shaking and then blocked for non‐specific staining using PBS containing 10% (v/v) goat serum. Coverslips were probed with the antibodies specified in the figure legends for 1 h at RT in PBS with 0.02% (w/v) sodium azide and 0.2% (w/v) BSA (wash solution). Coverslips were subsequently washed three times using wash solution for 10 min each and then incubated with Hoechst (DNA stain 1:10 000) containing secondary antibodies (1:500) conjugated to either Alexa Fluor 488 or 594 for 1 h. For lysotracker staining, cells were incubated with 50 nM of Lysotracker DeepRed (ex/em 647/668 nm) in serum‐free cell culture medium for 20 min prior to fixation with 4% (w/v) paraformaldehyde. For filipin staining (ex/em 360/480 nm), cells were fixed, washed briefly with glycine (1.5 mg/mL) and then washed three times with PBS before incubation with filipin at 50 μg/mL for 2 h. Coverslips were then rewashed three times with wash solution and placed face down onto glass microscope slides with a drop of ProLong Glass Antifade Mountant and sealed with nail varnish. Myoblasts were imaged using a Zeiss 710 microscope.

### Mitochondrial respiration

Mitochondrial bioenergetics were measured in L6 myoblasts using a Seahorse XF24 analyser. WT L6 muscle cells or those that had been genetically manipulated as specified in the figure legends were seeded and cultured in 24‐well Seahorse culture plates and switched to phenol‐ and serum‐free Dulbecco's modified Eagle's medium (DMEM) (standard AA formulation [#D5030‐10L]) containing 5 mM of glucose, 2 mM of glutamine and 10 mM of HEPES prior to analysis of basal respiration, protein synthesis‐dependent respiration, ATP‐linked respiration and non‐mitochondrial respiration (using cycloheximide, oligomycin, rotenone and antimycin). Live cell respiration parameters were normalized to cell protein.

### Isolation of lysosomal fractions

Lysosomal membranes were immunoprecipitated from myoblasts stably expressing the HA‐GFP‐TMEM192 protein. Control and HA‐GFP‐TMEM192 expressing cells were cultured in 15‐cm culture dishes, harvested and homogenized in a 2‐mL homogenizer. One hundred microlitres of homogenized lysates was transferred to 1.5 mL of Eppendorf tubes and was mixed with 10 μL of magnetic anti‐HA beads and left on a rotating wheel for 15 min at 4°C. The beads were subsequently collected using a magnet and washed three times using 500 mM of KCl prior to incubation with lysis buffer to detach magnetic beads from the HA tag.

### Amino acid uptake

AA uptake into myoblasts cultured on 12‐well plates was assayed using either [^3^H]‐leucine or [^14^C]‐Me‐AIB. Myoblasts were incubated in AA‐free EBSS for 2 h and subsequently washed twice with HEPES‐buffered saline prior to incubation in uptake buffer (containing 10 μM of [^3^H]‐leucine or [^14^C]‐Me‐AIB) for 10 min. Non‐specific tracer binding was quantified by determining cell‐associated radioactivity in the presence of a saturating dose of unlabelled Me‐AIB (10 mM) or leucine (20 mM). At the end of the 10‐min uptake incubation period, myoblasts were washed twice with ice‐cold 0.9% (w/v) sodium chloride prior to lysis with 1.25 mL of 50‐mM NaOH for 30 min. Cellular radioactivity in a 1‐mL lysate sample was quantified by liquid scintillation counting. The residual lysate was used for determining cell protein content by the Bradford method.

### Cholesterol measurement

Cholesterol content was measured using the Thermo Amplex Red cholesterol assay kit as per the manufacturer's instructions. Ten micrograms of lysosomal fractions was added to Amplex Red assay solution, which contains 300 μM of Amplex Red reagent, 2 U/mL of HRP as well as 2 U/mL of cholesterol oxidase, 0.2 U/mL of cholesterol esterase, 0.1 M of potassium phosphate, pH 7.4, 0.05 mM of cholic acid and 0.1% Triton X‐100. Samples were incubated with Amplex Red assay solution for 30 min at 37°C in the dark prior to monitoring fluorescence using a CLARIOstar plate reader set at excitation/emission wavelengths of 530/590 nm, respectively. The relative concentrations of cholesterol were calculated by reference to a standard curve generated using known concentrations of cholesterol. Cholesterol abundance was presented as nanograms of cholesterol per microgram of lysosomal protein, isolated by immunoprecipitation as described above.

### Statistical analysis

Statistical analysis was performed using GraphPad Prism using one‐way analysis of variance (ANOVA) and Tukey's post hoc test for multiple comparisons. For single comparisons, a paired (Student's) *t*‐test was used. Values were considered significantly different at a *P* value of <0.05.

## Results

### Caveolin‐3 is detectable in purified lysosomal membranes isolated from L6 muscle cells

**Figure 1:**
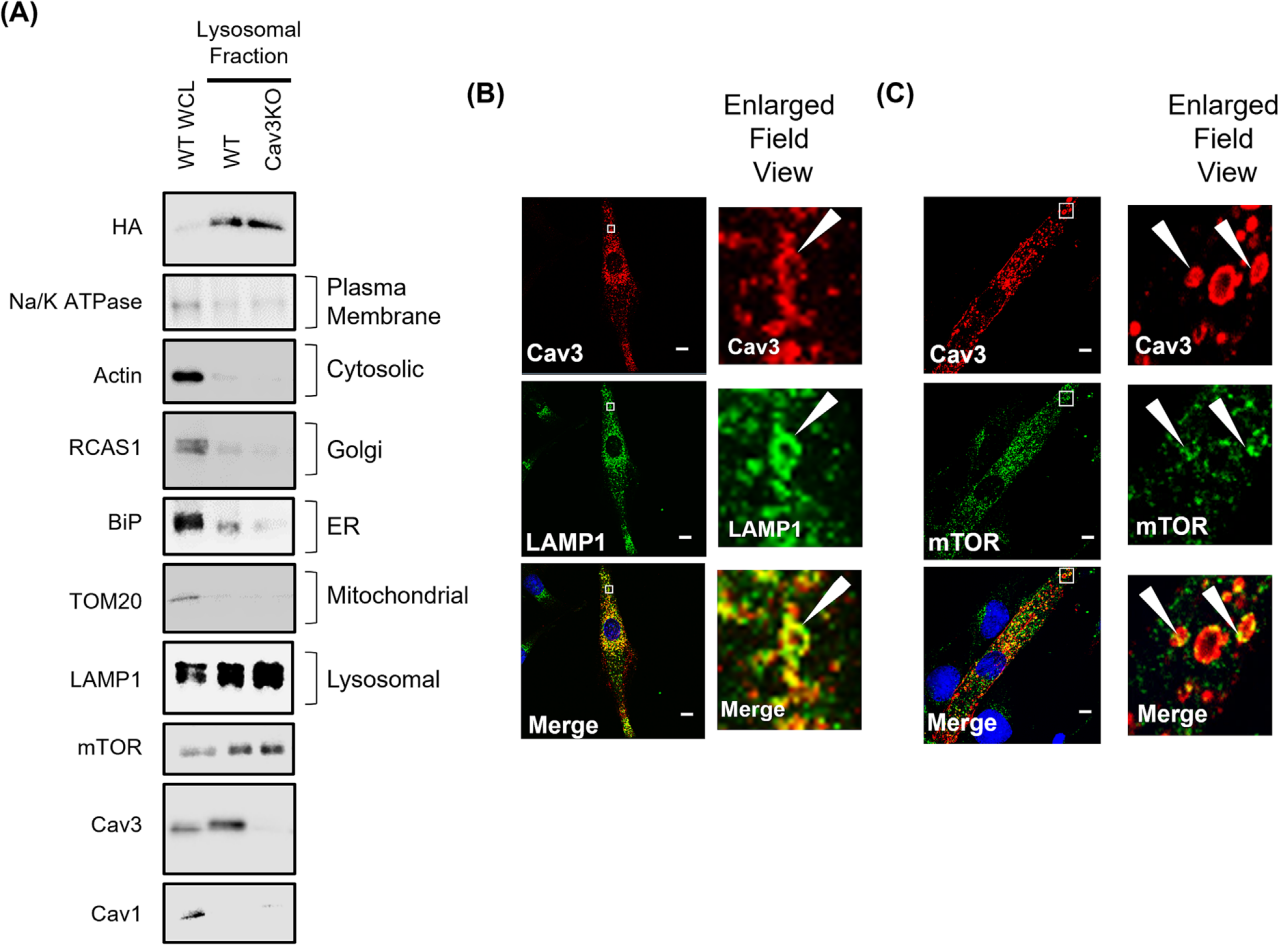
Caveolin‐3 (Cav3) is detectable in isolated lysosomal fractions. Lysosomal fractions from wild‐type (WT) or Cav3 knockout (Cav3KO) were isolated through the ‘pull‐down’ of HA‐labelled TMEM192 proteins as described in the Materials and methods section. Lysosomal fractions (10 μg of protein) from both WT and Cav3KO myoblasts were used for immunoblotting analysis with antibodies to proteins indicated (A) and compared with WT whole cell lysates (WCL). L6 muscle cells were fixed and stained with DAPI (nuclear blue staining) and immunostained for Cav3 (red) and lysosomal‐associated membrane protein 1 (LAMP1) (green) (B) or mTOR (green) (C). Potential regions of co‐localization are shown in the merged (yellow) images. Specific regions of interest within the white boxes are enlarged to highlight (white arrowheads) co‐localization. Scale bar = 10 μm.

Cav1 has previously been shown to localize at the lysosomal membrane in CHO cells where it has been linked to lysosomal cholesterol trafficking.^8^ To explore whether Cav3 might similarly associate with lysosomes, we isolated lysosomal membranes from L6 myoblasts. *Figure* *1A* shows that affinity purification of HA‐labelled lysosomal protein (TMEM192) resulted in the enrichment of the lysosomal marker LAMP1 and that of Cav3 compared with their immunoreactive abundance in whole cell lysates (*Figure* *1A*). As anticipated, Cav3 was not detectable in the lysosomal fraction prepared from myoblasts in which Cav3 expression had been deleted using CRISPR gene editing. It is noteworthy that the fractionation method resulted in very little contamination of the lysosomal fraction with marker proteins of the plasma membrane (Na/K ATPase), endoplasmic reticulum (ER) (BiP), mitochondria (TOM20) or Golgi (RCAS1). Whilst Cav1 has been shown to associate with lysosomal membranes from non‐muscle cell lines (CHO, U2OS or mouse embryonic fibroblasts [MEFs]), it was barely detectable in the lysosomal fraction prepared from L6 muscle cells (*Figure* *1A*). This latter finding is consistent with the view that Cav3 is the predominant Cav isoform in skeletal muscle tissue with Cav1 being expressed at much lower levels in this cell type. To further substantiate the potential association of Cav3 with the lysosome in L6 muscle cells, we used fluorescent microscopy to visualize the distribution of Cav3 and that of LAMP1. The fluorescent signals corresponding to Cav3 and LAMP1 overlap in multiple instances in ring‐like structures that are highlighted by arrowheads within the enlarged field views (*Figure* *1B*). Moreover, similar fluorescent staining revealed co‐localization between Cav3 and mTOR in L6 muscle cells (*Figure* *1C*). The observation that Cav3 closely associates with lysosomal membranes is not restricted to myoblasts, as we also observe immunocolocalization of Cav3 and LAMP1 signals in fully differentiated L6 myotubes by confocal microscopy (*Figure* *S1* *A*), using an antibody that specifically recognizes Cav3 (as highlighted by a lack of Cav3 staining in Cav3KO muscle cells) (*Figure* *S1* *B*).

### Basal and amino acid‐induced mTORC1 signalling is impaired in caveolin‐3‐depleted cells

**Figure 2:**
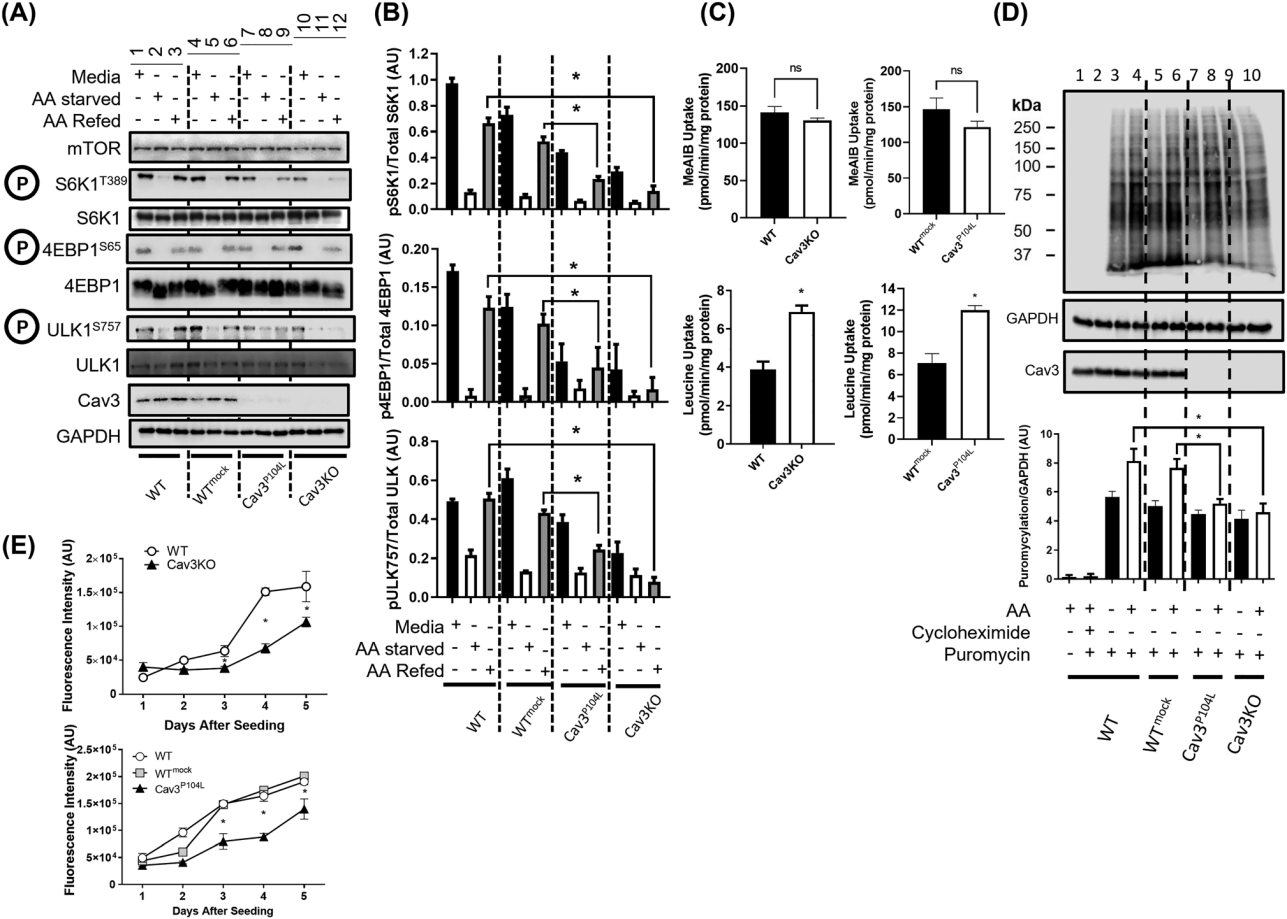
Amino acid‐induced mTORC1 activation, protein production and cellular proliferation are reduced in limb girdle muscular dystrophy type 1C (LGMD‐1C) and caveolin‐3 knockout (Cav3KO) muscle cells. Wild‐type (WT) L6 muscle cells or those stably transfected with an empty vector (mock), one encoding Cav3^P104L^ or had Cav3 deleted through gene editing using CRISPR/Cas9 (Cav3KO) were used for western blot analysis and DAPI proliferation assays. Whole cell lysates (WCL, 30 μg of protein) from WT, mock, Cav3^P104L^‐expressing and Cav3KO myoblasts were subject to sodium dodecyl sulfate‐polyacrylamide gel electrophoresis (SDS‐PAGE) and immunoblotting with antibodies targeting the phospho‐specific or native protein indicated during the treatments indicated (A) and phosphorylation of target proteins quantified from a minimum of three experiments relative to the abundance of the native protein (B). WT, mock, Cav3^P104L^ and Cav3KO cells were used to determine the cellular uptake of leucine or α‐methyl‐aminoisobutyric acid (Me‐AIB) and compared with WT L6 muscle cells (C). Muscle cells were incubated with puromycin or additionally pre‐incubated with cycloheximide and were subject to SDS‐PAGE and immunoblotting with antibodies against puromycin and GAPDH (D) and abundance of puromycylated proteins quantified from a minimum of three separate experiments relative to GAPDH (gel loading control) using ImageJ software. For DAPI proliferation assays, Cav3KO cells (upper panel) or cells expressing the P104L mutant (lower panel) were seeded at Day 1 and fixed using 4% (w/v) paraformaldehyde on the days indicated and subsequently stained with a nuclear DAPI stain where fluorescence was measured using a CLARIOstar plate reader (E). All graphical data represent mean ± SEM from a minimum of three separate experiments. Asterisks indicate a significant change (*P* < 0.05).

Given that we find Cav3 co‐localizes with both LAMP1 and mTOR (*Figure* *1B,C*) and activation of mTORC1 is widely considered to be initiated at the lysosomal membrane surface, the effect of Cav3 loss on nutrient (AA)‐dependent mTORC1 signalling was investigated. For this analysis, we compared the effects of AA withdrawal and resupplementation on phosphorylation of S6K1^Thr389^, 4EBP1^Ser65^ and ULK1^Ser757^ (all of which are downstream mTORC1 targets that play important roles in the regulation of protein synthesis and autophagy) in Cav3KO cells or muscle cells expressing the mutated Cav3^P104L^ protein with that seen in WT myoblast controls. As reported by us and others previously,^2^, ^7^ expression of Cav3^P104L^ results in substantial loss of native Cav3 within muscle cells (*Figures* *2A* and *S2* *B*) due to oligomerization of the latter with the mutant Cav3 and their targeted degradation by the ubiquitin–proteasome pathway. *Figure* *2A* shows that phosphorylation of S6K1^Thr389^, 4EBP1^Ser65^ and ULK1^Ser757^ in WT L6 myoblasts or those transfected with the EV (mock) used to introduce the Cav3^P104L^ construct was clearly detectable when muscle cells were maintained in media containing AAs (Lanes 1 and 4). The phosphorylation of all three proteins on their respective mTORC1 target sites diminished greatly when cells were AA starved for 2 h (Lanes 2 and 5) and, notably, recovered when starved cells were subsequently ‘AA refed’ (Lanes 3 and 6). The reduction and subsequent recovery in phosphorylation of these proteins is fully consistent with the inhibitory and stimulatory effects that AA withdrawal and resupplementation respectively have upon mTORC1 activity. Strikingly, muscle cells depleted of Cav3 (as a result of expressing the mutated Cav3^P104L^ or because of CRISPR gene editing) exhibited a marked reduction in basal S6K1^Thr389^, 4EBP1^Ser65^ and ULK1^Ser757^ phosphorylation when maintained in AA‐containing media (cf. Lanes 7 and 10 with Lane 1). Phosphorylation of these proteins was further reduced when cells were AA starved and, significantly, showed a poor ability to recover when AAs were resupplemented to incubation media (cf. Lanes 9 and 12 with Lanes 7 and 10, respectively) (AA‐stimulated phosphorylation status was also compared directly with AA‐depleted conditions in *Figure* *S3* *A*). It is important to stress that the reduced basal and AA‐stimulated phosphorylation of S6K1, 4EBP1 and ULK1 on their respective mTORC1 phosphorylation target sites (*Figure* *2A,B*) cannot be attributed a reduction in the cellular abundance of these proteins, which were unaffected by cellular loss of Cav3 expression. It is conceivable that the reduced capacity of AAs to promote mTORC1 activation may be a consequence of diminished recruitment/association of mTOR with lysosomal membranes. However, this seems unlikely given that we do not observe any notable loss in mTOR immunostaining at LAMP1‐positive membranes when comparing Cav3KO cells and WT myoblasts (*Figure* *S4*). It is also plausible that the observed reduction in mTORC1 activation within Cav3KO myoblasts might be a result of diminished AA uptake. To test this possibility, we measured the uptake of Me‐AIB, a paradigm substrate for the System A short‐chain SNAT2 neutral AA transporter, and that of leucine (an AA that displays primacy with respect to activation of the mTORC1/S6K1 axis). Whilst we saw no significant changes in System A transport activity, leucine uptake was notably increased in both Cav3KO and Cav3^P04L^ myoblasts compared with WT or mock‐transfected cells (*Figure* *2C*). These latter observations would imply that it is highly unlikely that the nutrient‐dependent reduction in mTORC1 signalling in Cav3‐deficient myoblasts can be linked to a reduction in AA delivery across the plasma membrane.

To understand the functional implications of the observed loss in mTORC1 signalling, we assayed the impact of Cav3 loss on protein synthetic capacity using the SUnSET method, which assays incorporation of puromycin into nascent polypeptides as a measure of global translation. *Figure* *2D* shows that the abundance of puromycylated proteins from Cav3^P104L^‐expressing or Cav3KO cells was significantly lower than that observed in control (WT or mock‐transfected) cells. Consistent with the reduced AA‐dependent phosphorylation of S6K1 and 4EBP1 seen in Cav3^P104L^ and Cav3KO cells (*Figure* *2A*), the ability of AAs to stimulate mRNA translation was also blunted in these cells as judged by the lower peptide puromycylation compared with AA‐treated control cells (*Figure* *2D*). As proliferating myoblasts would be highly dependent on protein synthesis, we assessed cellular proliferation in response to Cav3 depletion. *Figure* *2E* shows that proliferation of Cav3‐deficient myoblasts (Cav3^P104L^ or Cav3KO) was significantly reduced compared with that of WT cells or those expressing the EV (puromycylation under AA‐fed conditions was also directly compared with AA‐depleted conditions in *Figure* *S3* *B*).

### Respiration dedicated to protein synthesis is reduced in caveolin‐3 knockout myoblasts

**Figure 3:**
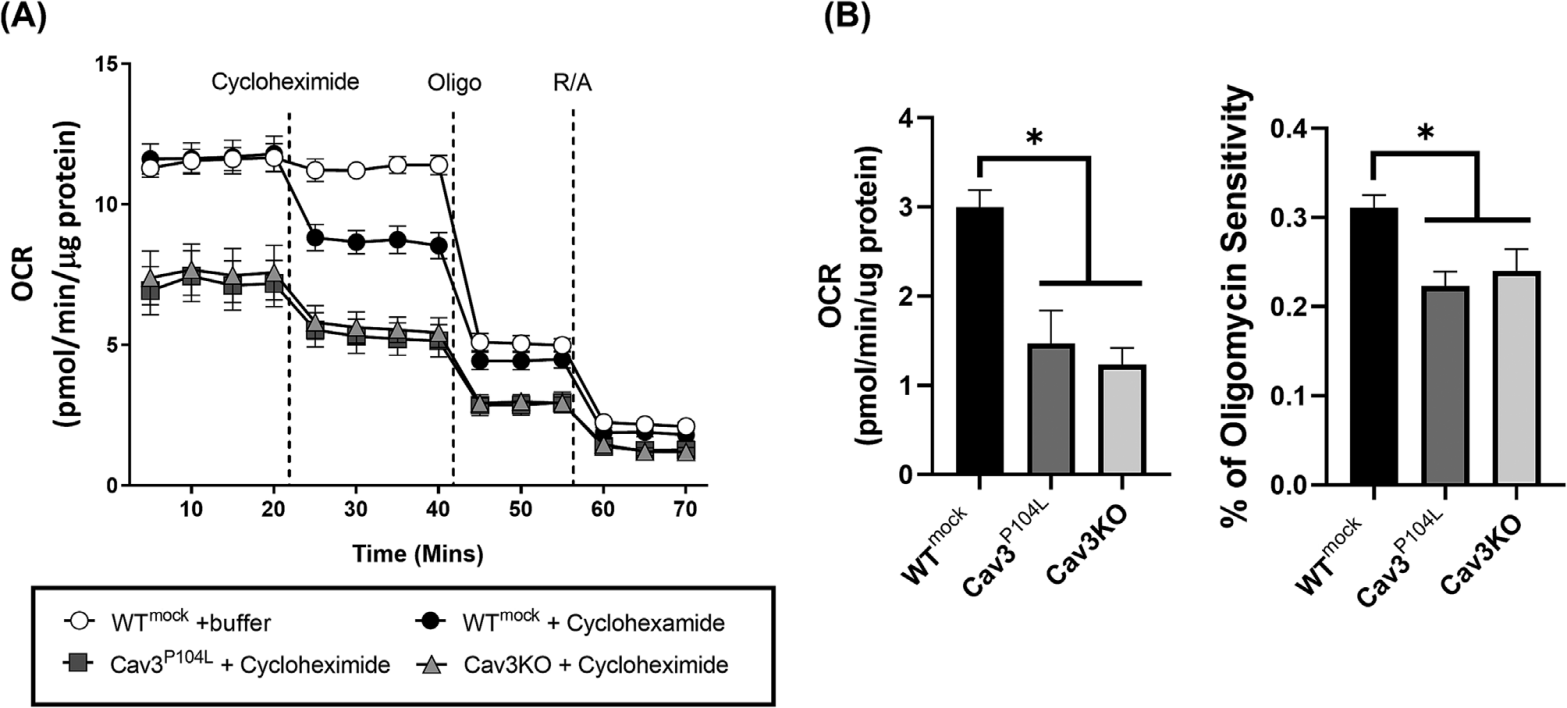
Respiration allocated to protein production is reduced in myoblasts depleted of caveolin‐3 (Cav3). Wild‐type (WT) L6 muscle cells stably transfected with an empty vector (mock), those expressing Cav3^P104L^ or muscle cells in which Cav3 has been genetically deleted by CRISPR/Cas9 gene editing (Cav3 knockout [Cav3KO]) were used for analysis of cell respiration using Seahorse technology. The oxygen consumption rate (OCR) trace shown in (A) is representative from a single experiment in which each point represents mean ± SEM of a triplicate measurement. Cycloheximide (1 μM), oligomycin (1 μM) and a mixture of rotenone (1 μM)/antimycin (2 μM) were added for determining how much of the cellular respiratory capacity (oxygen consumption) is allocated towards supporting protein synthesis (B). Bar graph data represent the analysis of a minimum of three individual experiments (values are mean ± SEM). Asterisks indicate significant change (*P* < 0.05) between bars specified.

Protein synthesis is an extremely energy‐dependent process and we have previously shown that Cav3‐depleted cells exhibit impaired mitochondrial morphology and ATP‐linked respiration.^7^ It is thus plausible that in addition to the reduction in mTORC1 signalling, the energy allocation for supporting protein synthesis is also impacted by cellular loss of Cav3. To test this proposition, we assayed cellular respiration and its sensitivity to cycloheximide (a protein synthesis inhibitor). *Figure* *3A/B* shows that the oxygen consumption rate (a measure of cell respiration) is lower in Cav3‐depleted muscle cells and, notably, the sensitivity to cycloheximide was significantly reduced compared with that observed in WT cells in which ~35% of ATP‐linked respiration was set aside for supporting protein synthesis (*Figure* *3B*).

### Analysis of caveolin‐3 knockout muscle

**Figure 4:**
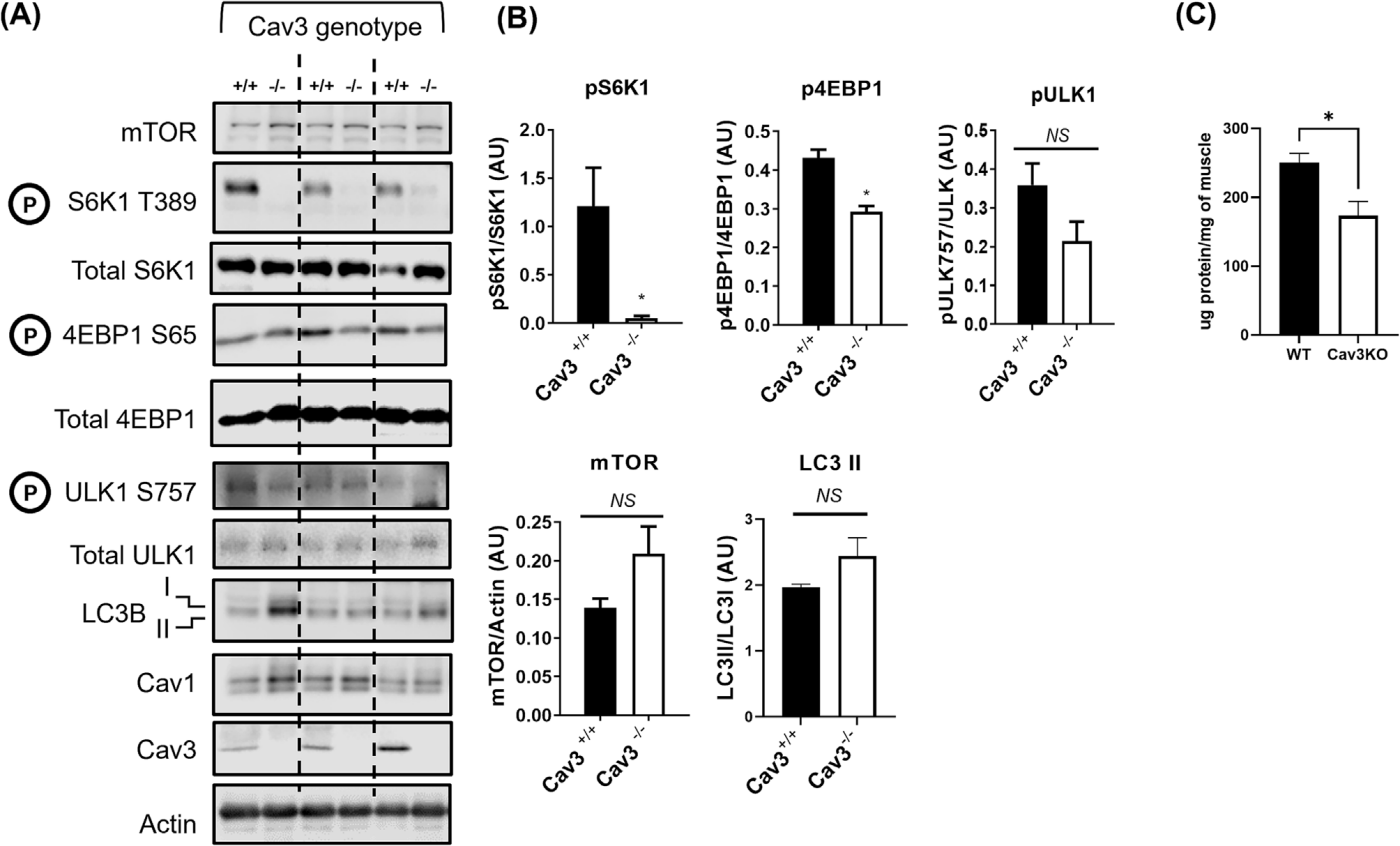
Effects of caveolin‐3 (Cav3) deficiency upon proteins linked to mTORC1 activity in skeletal muscle. Crude muscle homogenates prepared from gastrocnemius muscle of three wild‐type (WT, +/+) and three Cav3 knockout (Cav3KO, −/−) mice. Homogenates of mouse gastrocnemius were subjected to sodium dodecyl sulfate‐polyacrylamide gel electrophoresis and immunoblotted with antibodies to proteins shown (A) and protein abundance quantified relative to actin (used as a gel loading control) using ImageJ software (B). Total protein content of WT and Cav3KO mouse muscle was determined by using the Bradford assay and normalized against muscle mass used for protein determination (C). The data (mean ± SEM) are presented as fold change against WT muscle and are based on analysis of three WT and three Cav3KO mice. Asterisks indicate a significant change (*P* < 0.05).

Our cell‐based findings indicate that Cav3 deficiency is associated with a decline in mTORC1 signalling. To assess whether this may also be evident in vivo, crude muscle homogenates prepared from gastrocnemius muscle of fed WT^*(+/+)*^ and Cav3^*(−/−)*^ mice were used to evaluate phosphorylation of S6K1, 4EBP1 and ULK1. Strikingly, phosphorylation of S6K1 was reduced by ~95% despite no significant changes in mTOR abundance in Cav3^*(−/−)*^ muscle (*Figure* *4A/B*). 4EBP1 and ULK1, two additional downstream substrates of mTORC1, were also observed to display a fall in phosphorylation, although the reduction in ULK1 phosphorylation just failed to achieve statistical significance in the three separate mouse muscle preparations tested (*Figure* *4B*). It is noteworthy that the decline in S6K1, 4EBP1 and ULK1 phosphorylation in Cav3^*(−/−)*^ muscle could not be attributed to changes in the total intramuscular abundance of these proteins, which were unaffected. These observations suggest that, in line with our observations in L6 muscle cells, a reduction in mTORC1 signalling capacity is also a feature of Cav3‐deficient mouse muscle. We have previously noted that Cav3^−/−^ skeletal muscle exhibits reduced total muscle protein content when expressed per milligram of muscle. Consistent with that previous report and the reduced protein synthetic capacity we observe in Cav3KO‐depleted cells, *Figure* *4C* shows that the protein content in gastrocnemius muscle of Cav3^−/−^ mice was reduced by ~35% compared with that of WT mice.

### Manipulation of cholesterol content inhibits amino acid‐induced mTORC1 activation

**Figure 5:**
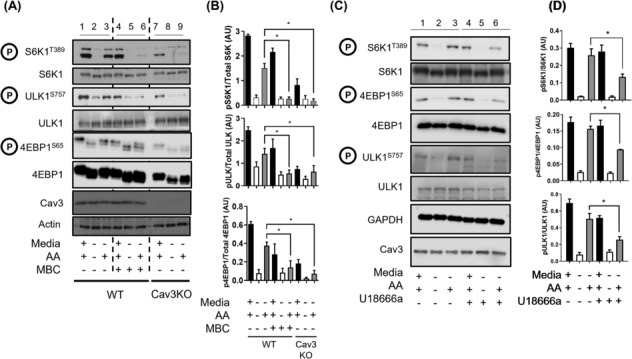
Effects of manipulating cellular cholesterol homeostasis in L6 muscle cells on amino acid (AA)‐induced mTORC1 activation. Wild‐type (WT) muscle cells were depleted of cholesterol for 2 h with 5 mM of methyl‐β‐cyclodextrin (MBC) or treated with the Niemann–Pick type C1 protein inhibitor U18666a (2.5 μg/mL) for 16 h prior to assessing AA‐dependent mTORC1 activation/phosphorylation of substrate proteins indicated in the figure. WT muscle cells or caveolin‐3 knockout (Cav3KO) muscle cells were held in AA‐containing media (Lanes 1, 4 and 7) or AA deprived for 2 h (Lanes 2, 5 and 8) or refed a physiological AA mix (20 min) after having been AA deprived (Lanes 3, 6 and 9) prior to cell lysis. Whole cell lysates (WCL, 30 μg of protein) were subject to sodium dodecyl sulfate‐polyacrylamide gel electrophoresis and immunoblotting with antibodies targeting the proteins indicated (A and C) and immunoreactive phospho (S6K1, ULK and 4EBP1) signals quantified relative to the native proteins from a minimum of three experiments using ImageJ software (B and D). All graphical data represent mean ± SEM from a minimum of three separate experiments. Asterisks indicate a significant change (*P* < 0.05).

As Cav proteins play an important role in cellular cholesterol trafficking, we entertained the possibility that disturbances in AA‐induced mTORC1 signalling associated with loss of Cav3 in muscle cells may partly arise because of changes in myocellular cholesterol homeostasis. To test this proposition, we incubated WT muscle cells with MBC, a cyclic oligosaccharide that sequesters and depletes cellular cholesterol,^22^ prior to subjecting cells to AA withdrawal/refeeding and analysis of mTORC1 signalling. *Figure* *5* shows the characteristic decline in phosphorylation of mTORC1 targets (S6K1, ULK1 and 4EBP1) when cells are AA starved and how acute resupplementation of AAs is associated with a robust increase in phosphorylation of all three mTORC1 target proteins (cf. Lanes 1–3). Significantly, however, whilst basal phosphorylation of S6K1, ULK1 and 4EBP1 was unaffected in muscle cells maintained in AA complete buffer upon cholesterol depletion with MBC, the ability to restate phosphorylation of these proteins in response to AA resupply following a 2‐h period of AA withdrawal was substantially reduced (*Figure* *5A*, cf. Lanes 3 and 6), mimicking the response seen in Cav3KO cells (*Figure* *5A*, cf. Lanes 6 and 9). As MBC treatment per se had no discernible impact on the abundance of S6K1, ULK1 and 4EBP1, the failure to reinstate their AA‐dependent phosphorylation in cholesterol‐depleted cells implies that maintaining the content of this sterol within key subcellular membrane compartments may be critical for supporting mTORC1 activation. Ratiometric analysis of the phosphorylation status of S6K1, ULK and 4EBP1 in the absence and presence of AAs in WT, MBC‐treated and Cav3KO myoblasts revealed a similar response pattern to that shown in *Figure* *5A*, underscoring that cholesterol depletion and loss of cellular Cav3 were associated with a marked reduction in AA‐dependent phosphorylation of the three mTORC1 target proteins (*Figure* *S3* *C*). It is worth highlighting that the ability to drive AA‐dependent mTORC1 activation following MBC treatment is not just compromised in L6 myoblasts but was also evident in a differentiated human skeletal muscle cell line, LHCN‐M2 (*Figure* *S5*).

Given the important role that lysosomes play in cholesterol homeostasis and in mTORC1 activation, the localization of Cav3 at this organelle may influence mTORC1 activation via modulation of lysosomal cholesterol. To gain further insight into this issue, we assessed the effect of U18666a, a pharmacological inhibitor of the Niemann–Pick type C1 protein (NPC1) that facilitates cholesterol efflux from the lysosome and whose inhibition results in accumulation of the sterol within the lysosomal lumen.^23^ *Figure* *5C* shows that whilst treatment of cells with U18666a in the presence of AAs did not significantly impact the basal phosphorylation of S6K1, 4EBP1 and ULK1, the capacity to phosphorylate these proteins in muscle cells that had been AA deprived and then resupplemented with AAs was significantly impaired compared with myoblasts not treated with U18666a (*Figure* *5D*, cf. Lanes 3 and 6, and *Figure* *S3* *D*).

**Figure 6:**
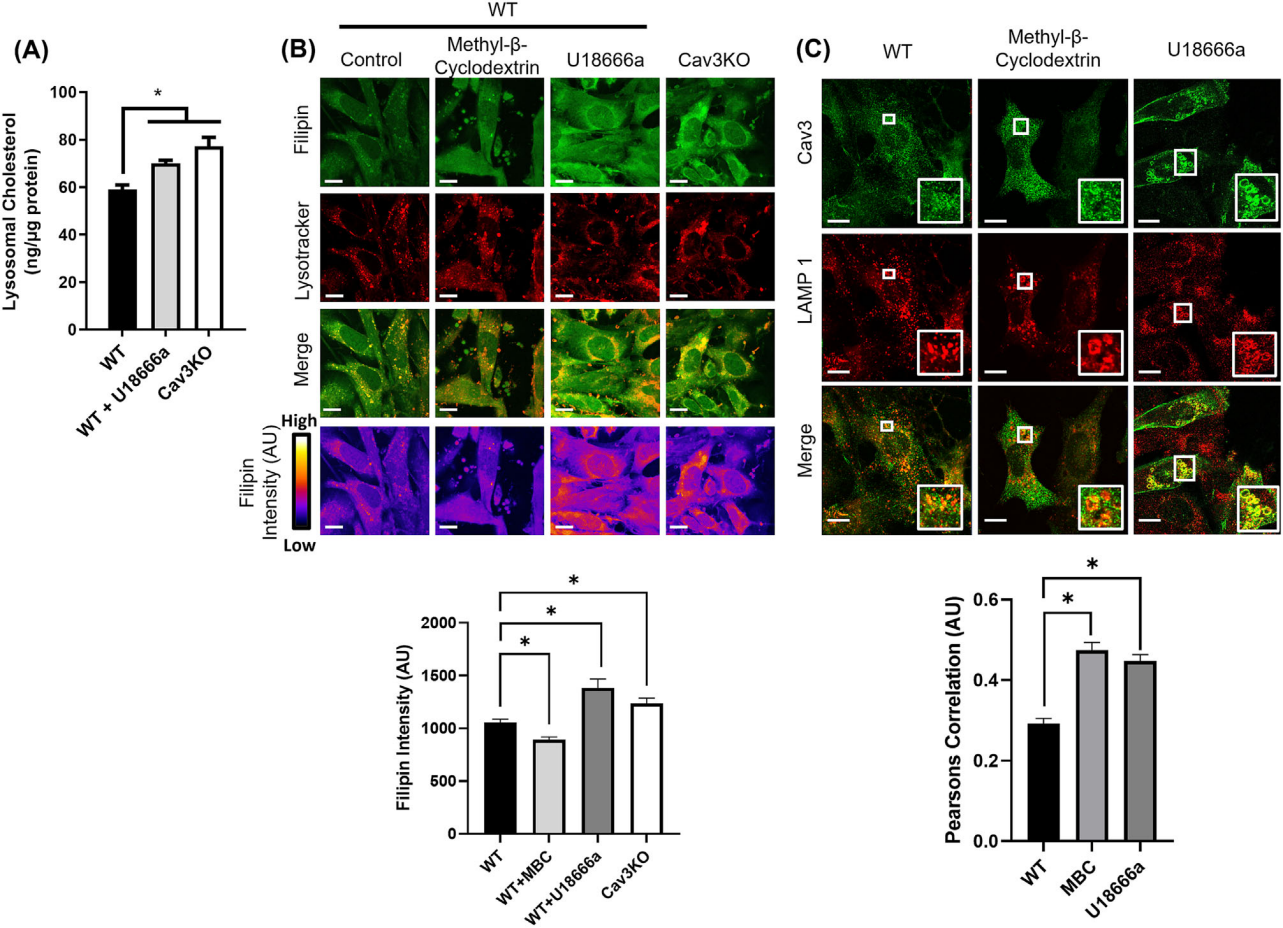
Lysosomal cholesterol levels are elevated in caveolin‐3 knockout (Cav3KO) L6 muscle cells and cholesterol manipulation enhances Cav3 co‐localization with lysosomal‐associated membrane protein 1 (LAMP1) in wild‐type (WT) muscle cells. Lysosomal fractions from WT L6 muscle cells, U18666a‐treated WT L6 cells or Cav3KO muscle cells were prepared by pull‐down of lysosomal HA‐labelled TMEM192 as outlined in the Materials and methods section and then used for quantification of cholesterol using the Thermo Amplex Red cholesterol quantification kit. Data are presented as nanograms of cholesterol per microgram of isolated lysosomal protein (A). WT L6 myoblasts, Cav3KO myoblasts or WT cells that were treated with methyl‐β‐cyclodextrin or U18666a were used for microscope analysis to assess filipin staining intensity. ImageJ was used to analyse microscope images and the ‘FIRE’ look‐up table (LUT) was used to display filipin intensity. Volocity software was used to determine the mean fluorescence intensity of filipin that intersected with lysotracker staining within confocal images (B). L6 muscle cells were fixed and immunostained for Cav3 (green) and LAMP1 (red) and overlayed (merge). Potential regions of co‐localization are shown in the merged (yellow) images. Specific regions of interest within the white boxes are enlarged to highlight co‐localization. The degree to which both signals co‐localize was assessed using Pearson's correlation (C). All graphical data represent mean ± SEM from a minimum of three images from four separate experiments. Asterisks indicate a significant change (*P* < 0.05). Scale bar = 10 μm.

Whilst MBC promotes non‐specific depletion of membrane cholesterol and U18666a induces cholesterol accumulation within the intra‐lysosomal compartment, both drugs promote a reduction in AA‐induced mTORC1 activation. As loss of mTORC1 signalling is also seen in Cav3‐depleted myoblasts, we asked whether this may be linked to changes in lysosomal cholesterol in Cav3KO myoblasts. To explore this possibility, L6 muscle cells stably expressing HA‐GFP‐tagged TMEM192 were used to affinity purify lysosomal fractions before and after overnight (16 h) treatment with U18666a followed by analysis of cholesterol content. *Figure* *6A* shows that NPC1 inhibition induced a significant (19%) increase in lysosomal cholesterol compared with that assayed in untreated myoblasts. Similar analysis within untreated Cav3KO cells revealed a 26% increase in lysosomal cholesterol. As a further means of assessing changes in lysosomal cholesterol in L6 myoblasts that had been treated with MBC and U18666a or in which Cav3 expression was deleted, filipin, a polyene antibiotic macrolide that fluoresces upon binding cholesterol, was used. L6 muscle cells were stained with lysotracker to visualize lysosomes before staining with filipin to visualize cholesterol. Cholesterol (filipin) staining that intersected with lysosomal staining was subsequently used as a readout to determine lysosomal cholesterol intensity/levels (*Figure* *6B*). Lysosomal cholesterol (filipin) was found to decrease modestly, but consistently when L6 muscle cells were pre‐treated with MBC and increased in myoblasts treated with U18666a or when assessed in Cav3KO cells (*Figure* *6B*). These observations indicate that Cav3 is likely to play a meaningful role in chaperoning/regulating lysosomal cholesterol and that disturbances in lysosomal cholesterol homeostasis may be a contributing factor impacting AA‐induced mTORC1 activation.

As Cav3 can localize at lysosomal membranes and Cav3 loss in L6 muscle cells impairs mTORC1 activation, we next sought to determine whether manipulation of cholesterol in L6 muscle cells affects association of Cav3 at the lysosome. For this, we assessed Cav3 and LAMP1 staining by confocal microscopy and performed correlation analysis of co‐staining within multiple images from separate experiments, which indicated that Cav3 association with the lysosome was significantly enhanced in muscle cells treated with either MBC or U18666a (*Figure* *6C*).

### Cellular re‐expression of caveolin‐3 in a caveolin‐3 null background mitigates the reduction in mTORC1 activation and protein synthesis

**Figure 7:**
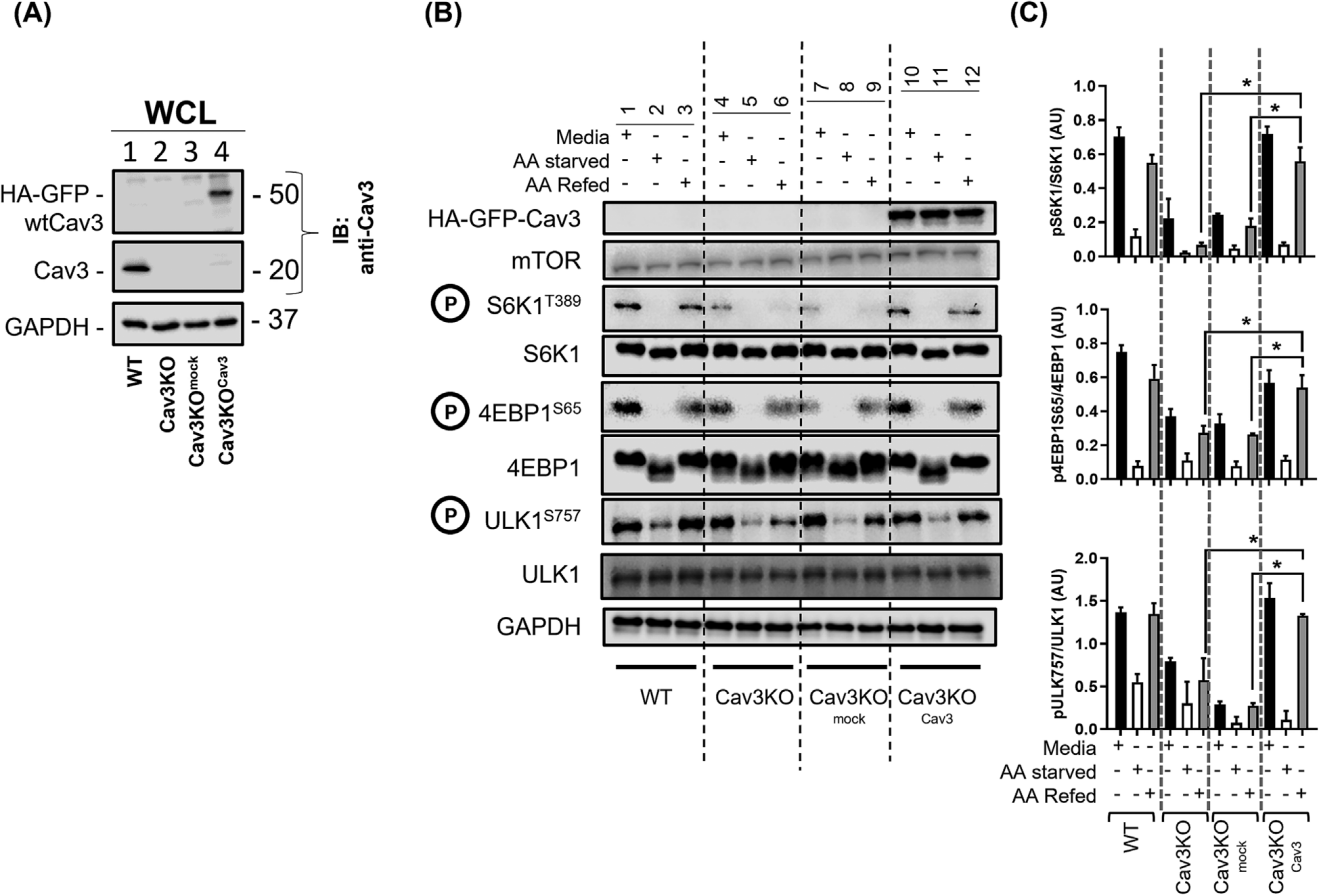
Re‐expression of caveolin‐3 (Cav3) restores mTORC1 signalling in Cav3 knockout (Cav3KO) muscle cells. Myoblasts in which Cav3 had been deleted by CRISPR/Cas9 (Cav3KO) were infected with retroviral particles containing an empty vector (Cav3KO + mock) or a vector containing HA‐GFP‐tagged wild‐type (WT)‐Cav3 (Cav3KO + Cav3) and stable transformants isolated by antibiotic selection prior to immunoblotting whole cell lysates (WCL, 30 μg of protein) with antibodies against Cav3 and GAPDH (used as a gel loading control) (A) or antibodies assessing phosphorylation of mTORC1 targets (pS6K1^T389^, p4EBP1^S65^ and pULK^S757^) and total S6K1, 4EBP1, ULK and GAPDH (B). Phosphorylation of proteins indicated was quantified from a minimum of three experiments relative to their total native protein content using ImageJ software (C). All graphical data represent mean ± SEM from a minimum of three separate experiments. Asterisks indicate a significant change between cell lines from the same day (*P* < 0.05).

As Cav3 loss was associated with a significant decline in AA‐induced mTORC1 signalling, we tested whether this could be mitigated by stable re‐expression of Cav3 in myoblasts that were Cav3 null. HA‐GFP‐tagged wild‐type Cav3 (HA‐GFP‐WT‐Cav3) was stably expressed in Cav3KO myoblasts. *Figure* *7A* shows that Cav3 antibodies were able to detect the HA‐GFP‐Cav3 fusion protein as a 50‐kDa immunoreactive band (Lane 4), but not in WT, Cav3KO or Cav3KO myoblasts that had been transfected with the vector lacking the fusion construct (Lanes 1–3). Notably, under similar exposure times, the intensity of the re‐expressed Cav3 fusion protein was very similar to that of the WT (~20 kDa) Cav3 band detected in WT myoblasts, suggesting that the abundance of the fusion protein in the null background was comparable with that of the native Cav3 protein in WT cells.

*Figure* *7B* shows the anticipated reduction and increase that AA withdrawal and resupply respectively have upon the phosphorylation of S6K1, 4EBP1 and ULK and that this is compromised in muscle cells lacking Cav3 (*Figure* *7B,C*). However, the re‐expression of Cav3 in Cav3KO myoblasts reinstated the ability of AAs to induce phosphorylation of these signalling molecules in myoblasts to a level comparable with that observed in WT cells (*Figure* *7C*).

**Figure 8:**
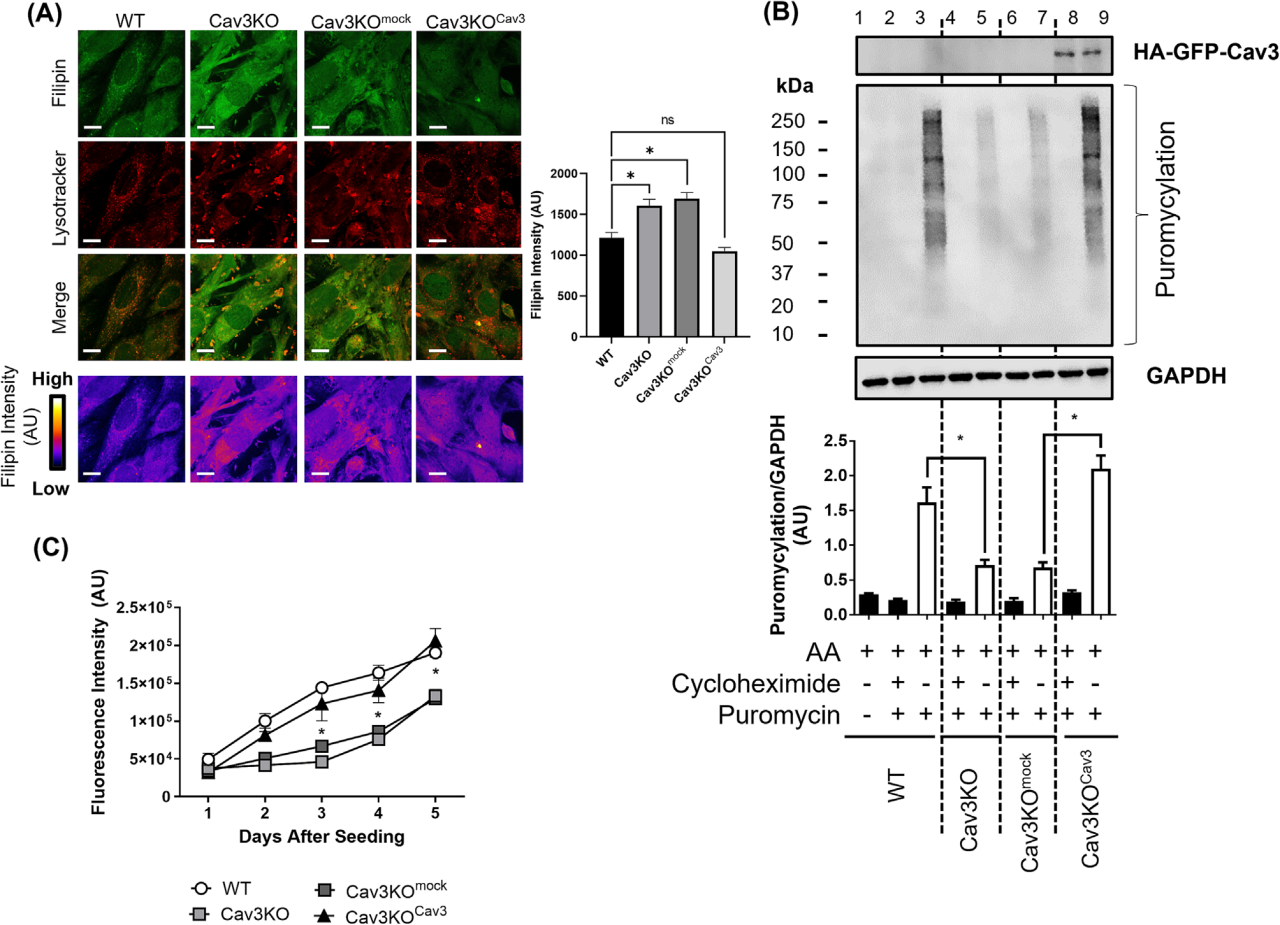
Re‐expression of caveolin‐3 (Cav3) mitigates changes in protein synthesis, lysosomal cholesterol and cell proliferation caused by myocellular Cav3 loss. Myoblasts in which Cav3 had been deleted by CRISPR/Cas9 (Cav3 knockout [Cav3KO]) were infected with retroviral particles containing an empty vector (Cav3KO + mock) or a vector containing HA‐GFP‐tagged wild‐type (WT)‐Cav3 (Cav3KO + Cav3), and stable transformants were isolated by antibiotic selection. L6 muscle cells were used for microscopy to assess cholesterol (filipin) intensity (A). Myoblasts were held in amino acid (AA)‐containing buffer prior to incubation with puromycin. In some experiments, muscle cells were pre‐incubated with cycloheximide before exposure to puromycin as indicated in the Materials and methods section. Muscle cells were lysed and protein puromycylation was assessed by sodium dodecyl sulfate‐polyacrylamide gel electrophoresis and immunoblotting with an anti‐puromycin antibody (B). Alternatively, the effects of re‐expressing Cav3 in Cav3‐deficient muscle cells on cell proliferation were assessed using a DAPI fluorescence‐based proliferation assay (C). All graphical data represent mean ± SEM from a minimum of three separate experiments. Asterisks indicate a significant change between cell lines from the same day (*P* < 0.05). Scale bar = 10 μm.

As re‐expression of Cav3 mitigated the reduction in mTORC1 signalling caused by its loss, we explored whether this might be linked to a normalization of lysosomal cholesterol content and, if so, whether this also had a corrective effect on key endpoints of mTORC1 signalling. *Figure* *8A* shows filipin and lysotracker staining and correlation analysis of their co‐staining to help define lysosomal cholesterol intensity. Whilst loss of Cav3 enhances lysosomal‐associated filipin staining, stable re‐expression of Cav3 resulted in lysosomal cholesterol labelling that was not too dissimilar from that seen in WT myoblasts. As Cav3KO myoblasts exhibited a decrease in de novo protein synthesis as assessed by the SUnSET puromycylation method (*Figure* *2D*), we investigated whether this too could be rescued by re‐expression of Cav3. Whilst Cav3KO cells or Cav3KO myoblasts transfected with an EV exhibit a marked reduction in puromycylated polypeptides (*Figure* *8B*, cf. Lanes 5 and 7 with 3), the expression of HA‐GFP‐WT‐Cav3 resulted in increased puromycylation of polypeptides that was comparable with that observed in WT cells (*Figure* *8A*, cf. Lane 9 with 3). Consistent with the effect that re‐expression of Cav3 in Cav3KO myoblasts had on AA‐stimulated mTORC1 signalling and protein synthetic capacity, we also observed a recovery in cellular proliferative capacity (*Figure* *8C*), which was comparable with WT L6 myoblasts.

## Discussion

The importance of lysosomes in cellular autophagy and the role they play in the activation of mTORC1 in response to nutrient and hormonal cues is now well established.^24^ mTORC1 activation by nutrients involves the orchestrated interplay of several proteins at the lysosomal membrane. For example, the v‐ATPase, which is required for establishing the acidification of the luminal compartment within the lysosome, has also been implicated in sensing AAs, possibly via its interaction with the Rag/Ragulator complex, and, as such, appears important in supporting mTORC1 activation.^25^ Whether the canonical acidification function of the v‐ATPase is necessary for mTORC1 activation is not entirely clear, but there is evidence that a low intra‐lysosomal pH per se is not a critical requirement.^25^ This latter observation may imply that the v‐ATPase has ancillary functions at the lysosome that include regulating the activation of the mTORC1 complex.^25^ Likewise, the lysosomal arginine transporter, SLC38A9, and the NPC1, which regulates the egress of cholesterol from the lysosome, have been implicated as protein components influencing the fidelity with which mTORC1 is activated.^18^ Whilst accepting that our experimental approach has involved global rather than lysosomal targeted depletion of Cav3, our findings provide strong support for the idea that Cav3 associates with the lysosomal compartment and may represent an additional component of the regulatory machinery supporting the nutrient‐dependent activation of the mTORC1 complex in skeletal muscle.

Previous studies in CHO cells and MEFs^8^, ^14^ demonstrated that Cav1 also localizes at the lysosomal membrane. Although the precise role of Cav1 at the lysosomes in these cells is poorly understood, it has been suggested that it may modulate lysosomal function and autophagy via regulation of autophagosome–lysosome fusion.^14^ Whilst autophagy or autophagosome–lysosome fusion was not a primary focus of the current study, we do observe increases in the lipidation of microtubule‐associated protein light chain 3 (LC3‐I) in Cav3‐depleted cells (*Figure* *S6*). LC3‐I lipidation or conversion to LC3‐II initiates the formation and lengthening of the autophagosome, which may be indicative of an increase in the initiation of autophagy within Cav3‐depleted muscle cells. Analysis of LC3‐II in Cav3^−/−^ gastrocnemius muscle also revealed a modest, albeit insignificant, increase (*Figure* *4*). However, whilst the increase in LC3‐II is fully consistent with our observed reduction in mTORC1 signalling, which would normally act to suppress autophagy, the increase in LC3‐II may also reflect a stall or failure in autophagic maturation that would be in line with the reduced mitophagy/autophagy that we and others have previously reported.^7^, ^26^ Moreover, the notion that autophagy may potentially be dependent upon Cav3 expression is supported by studies in cardiac muscle cells (HL‐1) in which Cav3 was found to interact with autophagy‐related proteins, beclin‐1 and Atg12, and influence their expression.^16^ Precisely how Cav3 abundance affects expression of such proteins and the autophagic process remains unclear, but addressing this issue remains an important investigative goal.

Precisely what regulates the dynamic nature of the interaction of Cav proteins with the lysosomal membrane is poorly understood. However, Cav proteins contain a cholesterol recognition amino acid census (CRAC) motif and readily bind the sterol on various intracellular membranes (e.g., lysosomes, mitochondria and ER) that is likely to facilitate chaperoning of cholesterol between different membrane compartments.^8^ The lysosomal interaction is considered transient as caveolins pick up cholesterol and traffic the sterol to other membranes within cavicles. This transient ‘kiss and run’ mechanism that has been suggested by Mundy and coworkers can be impaired when egress of lysosomal cholesterol is disrupted using inhibitors that target NPC1 (e.g., progesterone or U18666a) or those elevating lysosomal pH, which inhibits NPC2,^27^ a small globular protein involved in the transfer of cholesterol released from LDL to NPC1. In either case, inhibition of NPC1 or NPC2 results in the increased retention of cholesterol within the luminal compartment of the lysosome, and a concomitant increase in Cav1 at the lysosomal surface, whose residency may be ‘locked’ at this site because it is unable to access or extract the sterol for trafficking. A corollary to the above, as shown by Mundy et al.,^8^ is that a reduction in cholesterol within the lysosomal membrane, as would be expected by the chelating effect of cell treatment with cyclodextrin, would also promote an increase in Cav1 at the lysosomal membrane as it awaits ‘pick up’ from a cholesterol‐depleted membrane. In line with this idea, we show that inhibition of NPC1 with U18666a or cell treatment with MBC respectively increases and decreases lysosomal cholesterol and that, in either case, we observe increased localization of Cav3 at lysosomes within L6 myoblasts. The notion that Cav proteins are likely to be important for chaperoning cholesterol away from the lysosome to other membrane compartments is further strengthened by our finding that Cav3 deficiency in myoblasts increases lysosomal accumulation of the sterol, which can subsequently be reduced upon stable re‐expression of Cav3.

Notwithstanding the impact that myocellular Cav3 deficiency has upon lysosomal cholesterol content, we did not observe significant changes in lysosomal pH or lysosomal size as judged respectively by fluorescence activated cell sorting (FACS) analysis and comparative area analysis of lysotracker stained compartments within WT and Cav3KO myoblasts using ImageJ (*Figure* *S7*). Notably, however, Cav3 deficiency in both L6 myoblasts and gastrocnemius muscle from Cav3^−/−^ mice was associated with disturbances in mTORC1‐directed signalling as determined by reduced phosphorylation of three downstream targets, S6K1^T389^, 4EBP1^S65^ and ULK1^S757^. As reduced phosphorylation of these mTORC1 targets could not be explained by a decline in their cellular/tissue abundance or that of mTOR, the evidence supports the view that Cav3 expression and/or its localization at the lysosome in muscle cells is necessary for optimal mTORC1 activity/signalling. It is of interest to note, however, that Cav1, whose expression was either unchanged in Cav3‐deficient myoblasts or modestly elevated in gastrocnemius muscle of Cav3^−/−^ mice, was unable to functionally substitute for Cav3 in maintaining mTORC1 signalling in skeletal muscle (*Figures* *S2* *A* and *4*). This finding may simply reflect Cav3 primacy that is associated with it being the principal caveolin expressed in this tissue and, consequently, we cannot exclude the possibility that Cav1 may similarly exert a regulatory influence on mTORC1 signalling in other cell types where it is the primary isoform.

So how might Cav3 regulate nutrient‐dependent activation of mTORC1? There is substantial evidence in the literature highlighting how caveolins and their localization within membrane caveolae serve as a nexus for regulating diverse signalling pathways initiated by receptors that coalesce within caveolar domains where their activity can be modulated by direct interaction with resident Cav proteins (see previous study^28^). This interaction need not be restricted to cell surface caveolae and can also occur on other membranes where Cav proteins assemble.^29^ The ability to interact with signalling proteins is thought to be mediated by a 20‐AA sequence within caveolins known as the caveolin scaffolding domain (CSD) and an aromatic‐rich caveolin binding motif (CBM) within its binding partners.^30^ It is possible that Cav3 may function as a scaffolding molecule around which components involved in mTORC1 signalling may assemble. This notion is partly supported by in silico analysis of numerous signalling molecules associated with mTORC1 activation in which CBMs have been identified, including mTOR, raptor, Rag proteins, the C1 subunit of the v‐ATPase, NPC1 and the lysosomal AA transporter SLC38A9 (SNAT9) (*Figure* *S8*). Interestingly, Cav1 was identified as one of many proteins to interact with the N‐terminal fragment of SLC38A9 (AAs 1–119) when the latter was used as bait in coimmunoprecipitation and mass spectrometric‐based proteomic studies, raising the possibility that these two proteins may interact in vivo.^31^ The N‐terminal fragment of SLC38A9 is considered critical for association of the AA transporter with the Ragulator and for activation of mTORC1 at the lysosome. Whilst this specific region of SLC38A9 does not appear to harbour any discernible CBMs, such motifs are present in both the cytoplasmic and luminal facing domains of the transporter. Whether these CBMs are functionally important for AA sensing and signalling is currently unknown, but, if they are, it is possible that Cav3 may interact with the transporter and other mTORC1 signalling components to help stabilize and/or retain the complex for activation at the lysosome.

A more plausible explanation that may account for the disturbances in mTORC1 signalling is one that links Cav3 loss to changes in lysosomal cholesterol, which have already been alluded to in the discussion above. The export of cholesterol from the lysosome involves the tandem transfer of the bound sterol from NPC2 to NPC1.^32^ Previous work has identified a CBM within NPC1 that is directly adjacent to its sterol sensing domain and demonstrated that Cav1 and NPC1 can be coprecipitated from human fibroblasts.^33^ Significantly, the latter study showed that silencing Cav1 increased LDL‐derived cholesterol in late endocytic/lysosomal compartments, indicating that the NPC1 and Cav1 interaction is important in supporting cholesterol efflux from these compartments.^33^ This interaction may also be relevant to our understanding of how Cav proteins might impact on mTORC1 signalling. Castellano et al. have suggested that LDL‐derived cholesterol may influence the activation status of mTORC1 via the regulatory inputs the signalling complex receives from its interaction with NPC1 and SLC38A9, which may function as cholesterol sensors.^18^ These authors suggest that SLC38A9 conveys lysosomal cholesterol sufficiency via its conserved sterol‐interacting motifs to positively enhance mTORC1 activation. In contrast, it has been suggested that the association of NPC1 with the mTORC1 complex translates cholesterol insufficiency into mTORC1 inhibition and that deletion of NPC1 renders the signalling complex resistant to inhibition under circumstances of sterol depletion.^18^ This proposed model has now been further refined by the same group to include a new player in the sterol‐dependent regulation of mTORC1 signalling. Shin et al. recently implicated a cholesterol binding GPCR‐like protein (LYCHOS) that, in response to cholesterol sufficiency, sequesters the GTPase, GATOR1, from targeting RagA‐bound GTP that crucially supports activation of mTORC1.^34^

Whilst the involvement of LYCHOS adds a further layer to our understanding of the sterol‐dependent regulation of mTORC1, there is also a view that cholesterol trafficking across the limiting membrane of the lysosome may, itself, be the critical stimulus influencing mTORC1 activation. This proposition is based on the finding that the effects of itraconazole, a drug that blocks cholesterol trafficking through lysosomes, cause inhibition of mTOR activity in endothelial cells and that this can be mitigated by thapsigargin (which restores lysosomal cholesterol transport to the plasma membrane by promoting ER calcium release) or by exogenous provision of cholesterol/cyclodextrin to cells in which NPC1 or intracellular cholesterol trafficking has been inhibited using U18666a or imipramine, respectively.^35^ As Cav3 deficiency in myoblasts mimics the effects of NPC1 inhibition on lysosomal cholesterol content (*Figure* *6*) and dysfunctional endosomal/lysosomal calcium homeostasis is a feature of NPC1‐mutant cells,^36^ an important investigative goal of future studies will be to assess whether mobilizing ER calcium to help reinstate cholesterol trafficking also helps counter the loss in nutrient‐dependent mTORC1 signalling seen in Cav3‐deficient myoblasts.

The importance of mTORC1 as a regulator of metabolism, protein synthesis and cellular growth is well established.^37^ Consequently, a decline in mTORC1 signalling as seen in muscle cells lacking functional Cav3 may be an important contributing factor in the pathology of LGMD‐1C, which is characterized by a reduction in muscle fibre size because of their degeneration and/or necrosis.^2^ The maintenance and growth of muscle is a highly energetic process and one dependent upon regulation of processes contributing to protein turnover. The energy costs associated with protein turnover are not negligible and our work reveals that in WT L6 muscle cells, over one third of the cellular respiratory capacity is allocated for protein translation, whereas, by comparison, this allocation was notably reduced in Cav3‐depleted (Cav3^P104L^ or Cav3KO) myoblasts. This energy deficit may, in part, be attributed to the profound negative effect that Cav3 loss has on mitochondrial form and function. We have previously reported that Cav3 depletion results in significant impairment in mitochondrial respiratory function, which is associated with reduced expression of proteins essential for respiratory chain function (succinate dehydrogenase), reactive oxygen species neutralization (MnSOD), mitochondrial fusion (MFN2 and OPA1) and mitochondrial biogenesis (PGC1α).^7^ This reduced respiratory capacity results in diminished ATP synthesis and a consequential lowering of the ATP:ADP ratio. One expectation of this reduced energy balance would be increased activation of AMP‐activated protein kinase (AMPK), widely regarded as a cellular energy sensor that ‘switches off’ energy consuming processes whilst stimulating those that would help restore the ATP energy reserve.^38^ Significantly, AMPK can also localize at the lysosomal membrane where its activation by the AXIN/LKB1 complex promotes interaction with and/or changes the phosphorylation/activity of proteins implicated in mTORC1 regulation (e.g., TSC2, Raptor, LAMTOR1 and other members of the Ragulator) in a manner that promotes dissociation of mTORC1 from the lysosome and inhibition of mTORC1 signalling.^17^ Given that the AMPK and TOR pathways are interlinked, it is highly likely that inhibition of mTORC1 in Cav3‐depleted myoblasts is driven not only by disturbances in lysosomal cholesterol trafficking but also by dysregulation of energy metabolism. This latter supposition is supported by our finding that AMPK is activated within Cav3KO myoblasts based on the elevated phosphorylation of its downstream physiological target, acetyl‐CoA carboxylase (*Figure* *S6* *B*).

In summary, our work reveals that Cav3 associates with lysosomal membranes and that loss of Cav3 induced by myocellular expression of the LGMD‐1C Cav3^P104L^ mutation or targeted Cav3 gene deletion in vivo or in vitro is associated with a reduction in mTORC1 signalling capacity, which we suggest is likely linked to changes in lysosomal cholesterol content/trafficking. The decline in mTORC1 signalling seen in Cav3‐depleted myoblasts is associated with a reduction in myocellular protein synthesis and cell growth, which we find is countered upon re‐expression of Cav3 that not only helps to restate mTORC1‐directed signalling but also mitigates changes in lysosomal cholesterol. Notwithstanding the limitations of the cell‐based studies presented here, we and others have previously indicated that Cav3 loss associated with expression of the LGMD‐1C Cav3^P104L^ mutation results in poor fusion and differentiation of myoblasts and retention of an immature cell signature that may contribute to the myopathic changes in muscle fibre size and necrosis seen in LGMD‐1C patients.^7^, ^39^ Whether these myopathic changes are partly driven by disturbances in lysosomal trafficking of cholesterol and mTORC1 signalling in skeletal muscle of LGMD‐1C patients is currently unknown. However, recent work demonstrating increased accumulation of cholesterol in skeletal muscle of Duchenne muscular dystrophy patients and mdx mice, as well as the finding that pharmacological normalization of cholesterol content improved dystrophic parameters in mdx dystrophic muscle, raises the interesting possibility that impaired intramuscular cholesterol homeostasis may potentially serve as a common trigger of the muscle atrophy process.^40^ If so, modulation of cholesterol metabolism and trafficking in skeletal muscle may represent an important therapeutic target for the treatment and management of muscular dystrophies.

## Conflict of interest statement

The authors confirm that there are no conflicts of interest to declare.

## Supporting information


**Figure S1:** A Cav3 specific antibody co‐localises Cav3 with LAMP1 in differentiated L6 myotubes. Myoblasts were grown to 70% confluence in cell culture dishes on 13 mm coverslips and fixed or differentiated to form multinucleated myotubes prior to being fixed using 4% (w/v) paraformaldehyde. WT myotubes (A) and WT or Cav3KO myoblasts (B) were then probed with both Cav3 and LAMP1 antibodies, prior to incubation with secondary antibodies and visualised using a Zeiss 710 microscope. Overlapping Cav3 (green) and LAMP1 (red) signals appear in yellow in the merged panel. Scale bar = 10 μm.

**Figure S2:** Confirmation of Cav3 depletion by CRISPR/Cas9 and by introduction of the Cav3^P104L^ mutant. Immunoblot analysis of L6 muscle cells in which Cav3 expression has been deleted by use of CRISPR/Cas9 gene editing (A), or in which WT L6 cells express an empty vector or a vector encoding the Cav3^P104L^ (B). Whole cell lysates (30 μg protein) were used for immunoblot analysis and probed with antibodies against proteins shown.

**Figure S3:** Ratiometric analysis of S6K1, 4EBP1 and ULK1 phosphorylation assessed in the absence and presence of amino acids in Cav3 depleted cells or in cells in which cholesterol was pharmacologically manipulated. The degree to which amino acids induce phosphorylation of mTORC1 substrates, S6K1, 4EBP1 or ULK1 in response to Cav3 depletion (A), methyl‐β‐cyclodextrin (5 mM for 2 h) (C), U18666a treatment (2.5 μg/ml for 16 h) (D) or puromycin incorporation into nascent polypeptides (B) was compared directly with amino acid starved conditions in the indicated cell lines.

**Figure S4:** Association of mTOR with LAMP1 in WT and Cav3KO myoblasts. Wild‐type and Cav3KO myoblasts transfected with ds‐LAMP1 were grown on 13 mm coverslips were fixed and probed with the mTOR antibody as described in the methods section. Pearsons Correlation was used to determine the degree of co‐localisation between mTOR and dsred‐LAMP1 where *ns* >0.05. Scale bar = 10 μm.

**Figure S5:** Amino acid stimulated mTORC1 signalling is impaired when lysosomal cholesterol content is manipulated in LHCN‐M2 human muscle cells. Human LHCN‐M2 skeletal muscle cells (grown in DMEM/M199 medium (4:1) supplemented with penicillin streptomycin (100 μg/ml), FBS 15% (v/v) HEPES (20 mM), Zinc sulphate (30 ng/ml), vitamin B12 (1.4 μg/ml), dexamethasone (55 ng/ml), hepatocyte growth factor, recombinant human (2.5 ng/ml), and basic FGF (10 ng/ml)) were cholesterol depleted using 5 mM methyl‐β‐cyclodextrin for 2 h or pre‐treated with U18666a at 2.5 μg/mL for 16 h prior to serum and amino acid starvation using EBSS for 2 h. Muscle cells were subsequently refed with AA for 20 min.

**Figure S6:** Assessment of microtubule‐associated protein light chain 3 (LC3) lipidation and AMPK activation LC3 lipidation (LC3II) was assessed and compared to relative non‐lipidated LC3 (LC3I) and used as a readout for autophagic flux in WT or WT^mock^ muscle cells compared with Cav3KO or Cav3^P104L^ expressing cells respectively. Phosphorylation of an AMPK substrate Acetly CoA Carboxylase (pACC^Ser79^) was assessed in response to Cav3 depletion.

**Figure S7:** Lysotracker staining in wild type L6 muscle cells or muscle cells deficient in Cav3. Wild type (WT) L6 myoblasts, Cav3KO L6 myoblasts, L6 myoblasts in which an empty vector or the Cav3^P104L^ mutant was expressed were used for FACS analysis, microscopy or microplate readings. Lysosomal acidification was determined using FACS analysis of WT and Cav3KO myoblasts by assessing Lysotracker Deepred staining before or after pre‐treatment of cells with 200 nM of Bafilomycin. Following treatment muscle cells were fixed with 4% (w/v) PFA prior to analysis at excitation/emission wavelengths of 647/668 nm and analysis of mean geometric fluorescence intensity (A) or imaging using confocal microscopy (B/C). Volocity software was used to determine the mean fluorescence intensity from Lysotracker stained cells within confocal images (B) or lysosomal diameter (C). Alternatively, a plate reader was used to determine fluorescence intensity of Lysotracker stained cells (D). Bar graphs represent triplicate mean values ± SEM where *NS* indicates no significant difference.

**Figure S8:** *In Silico* identification of caveolin binding motifs (CBM) within proteins involved in lysosomal cholesterol homeostasis and mTORC1 activation. Caveolin binding motifs (CBM) (A) were identified by *in silico* analysis in a number of proteins implicated in the activation of mTORC1 (B).
