# Supplementary figures and images for: Caveolin‐3 loss linked with the P104L LGMD‐1C mutation modulates skeletal muscle mTORC1 signalling and cholesterol homeostasis
Source: J Cachexia Sarcopenia Muscle. 2023 Sep 6;14(5):2310–26. doi: 10.1002/jcsm.13317 (PMC10570080; doi:10.1002/jcsm.13317)

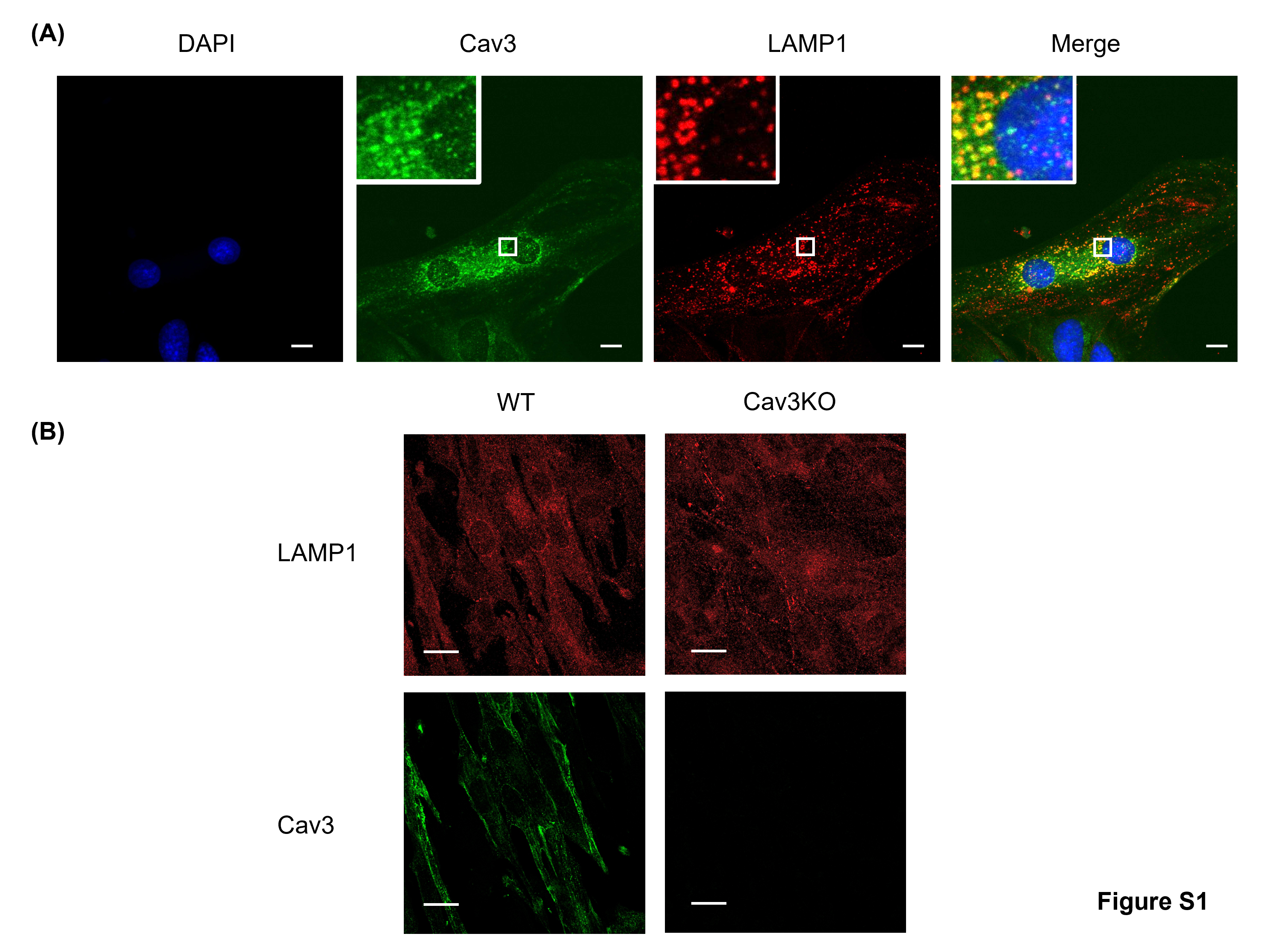

Supplement: Supplementary file 1 — Figure S1: A Cav3 specific antibody co‐localises Cav3 with LAMP1 in differentiated L6 myotubes. Myoblasts were grown to 70% confluence in cell culture dishes on 13 mm coverslips and fixed or differentiated to form multinucleated myotubes prior to being fixed using 4% (w/v) paraformaldehyde. WT myotubes (A) and WT or Cav3KO myoblasts (B) were then probed with both Cav3 and LAMP1 antibodies, prior to incubation with secondary antibodies and visualised using a Zeiss 710 microscope. Overlapping Cav3 (green) and LAMP1 (red) signals appear in yellow in the merged panel. Scale bar = 10 μm. [file JCSM-14-2310-s002.tif]

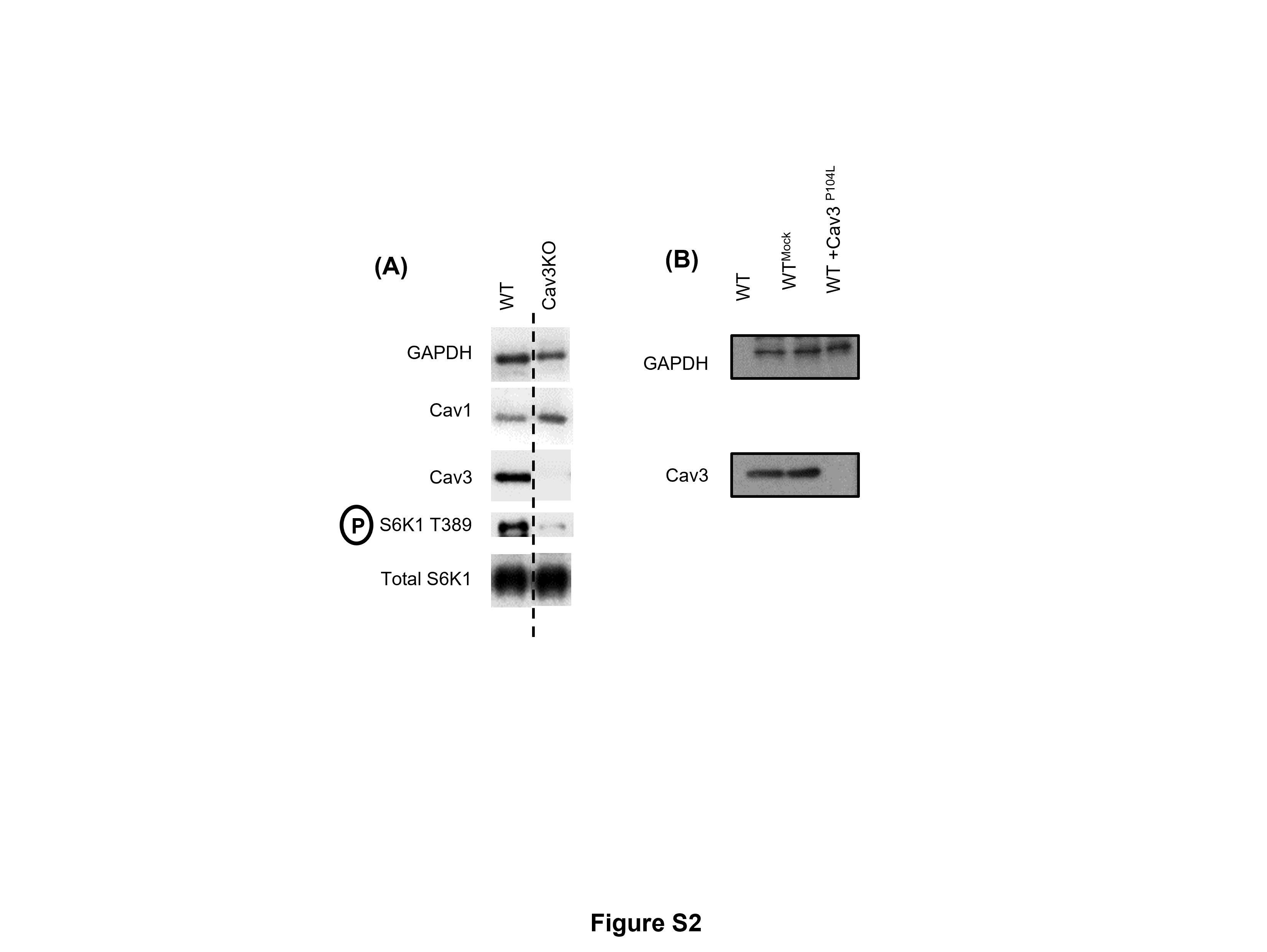

Supplement: Supplementary file 2 — Figure S2: Confirmation of Cav3 depletion by CRISPR/Cas9 and by introduction of the Cav3P104L mutant. Immunoblot analysis of L6 muscle cells in which Cav3 expression has been deleted by use of CRISPR/Cas9 gene editing (A), or in which WT L6 cells express an empty vector or a vector encoding the Cav3P104L (B). Whole cell lysates (30 μg protein) were used for immunoblot analysis and probed with antibodies against proteins shown. [file JCSM-14-2310-s006.tif]

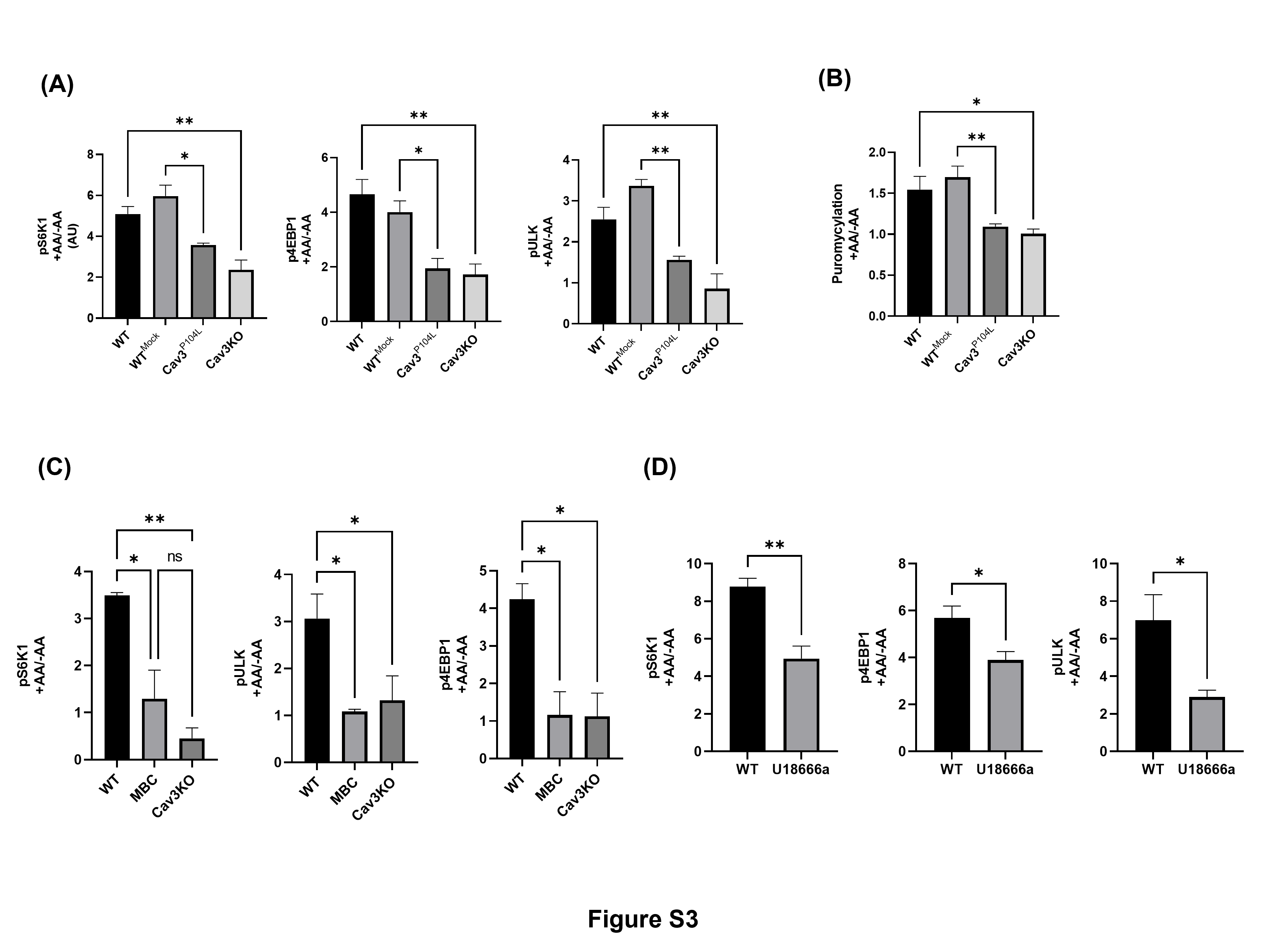

Supplement: Supplementary file 3 — Figure S3: Ratiometric analysis of S6K1, 4EBP1 and ULK1 phosphorylation assessed in the absence and presence of amino acids in Cav3 depleted cells or in cells in which cholesterol was pharmacologically manipulated. The degree to which amino acids induce phosphorylation of mTORC1 substrates, S6K1, 4EBP1 or ULK1 in response to Cav3 depletion (A), methyl‐β‐cyclodextrin (5 mM for 2 h) (C), U18666a treatment (2.5 μg/ml for 16 h) (D) or puromycin incorporation into nascent polypeptides (B) was compared directly with amino acid starved conditions in the indicated cell lines. [file JCSM-14-2310-s004.tif]

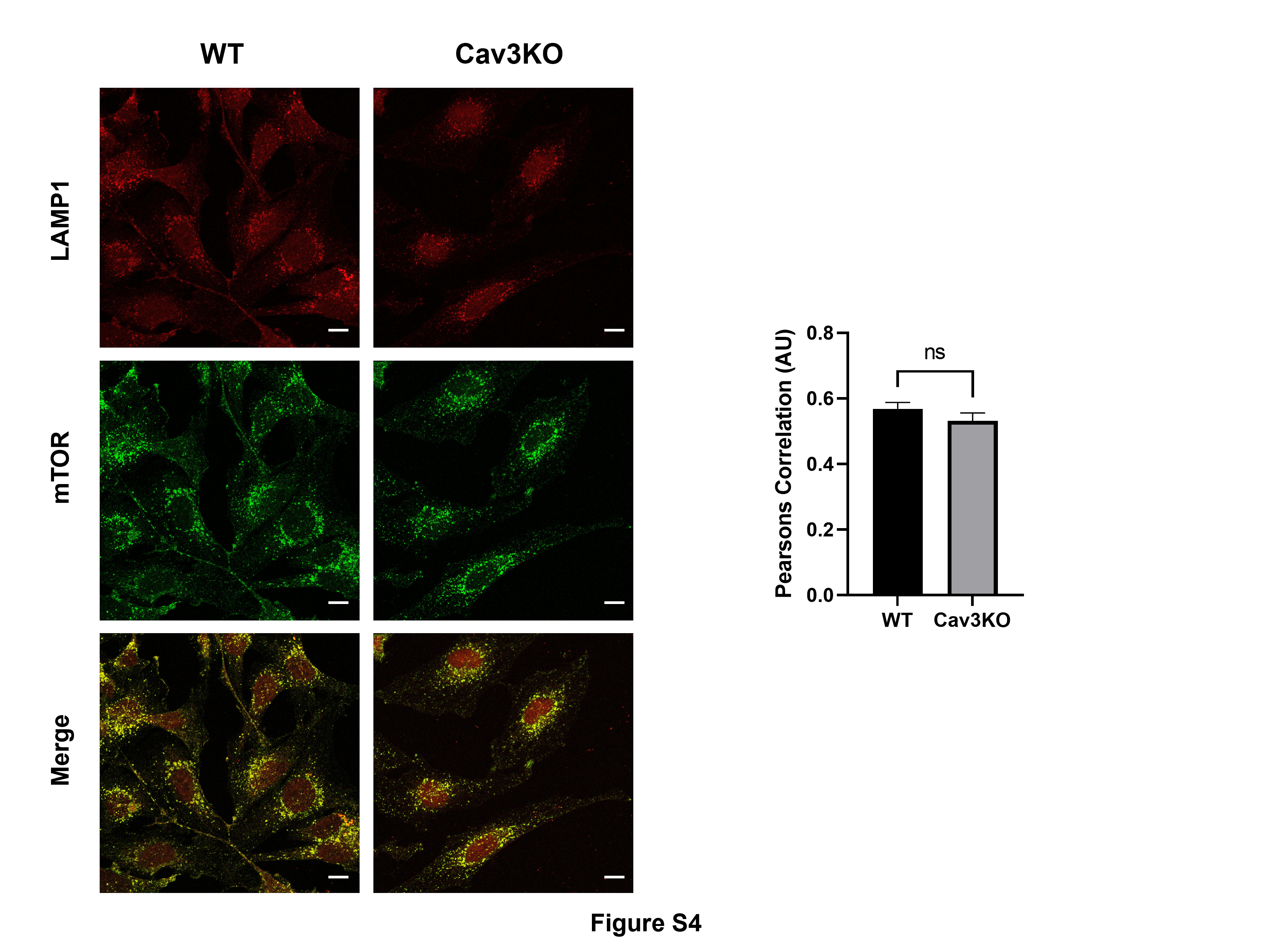

Supplement: Supplementary file 4 — Figure S4: Association of mTOR with LAMP1 in WT and Cav3KO myoblasts. Wild‐type and Cav3KO myoblasts transfected with ds‐LAMP1 were grown on 13 mm coverslips were fixed and probed with the mTOR antibody as described in the methods section. Pearsons Correlation was used to determine the degree of co‐localisation between mTOR and dsred‐LAMP1 where ns >0.05. Scale bar = 10 μm. [file JCSM-14-2310-s001.tif]

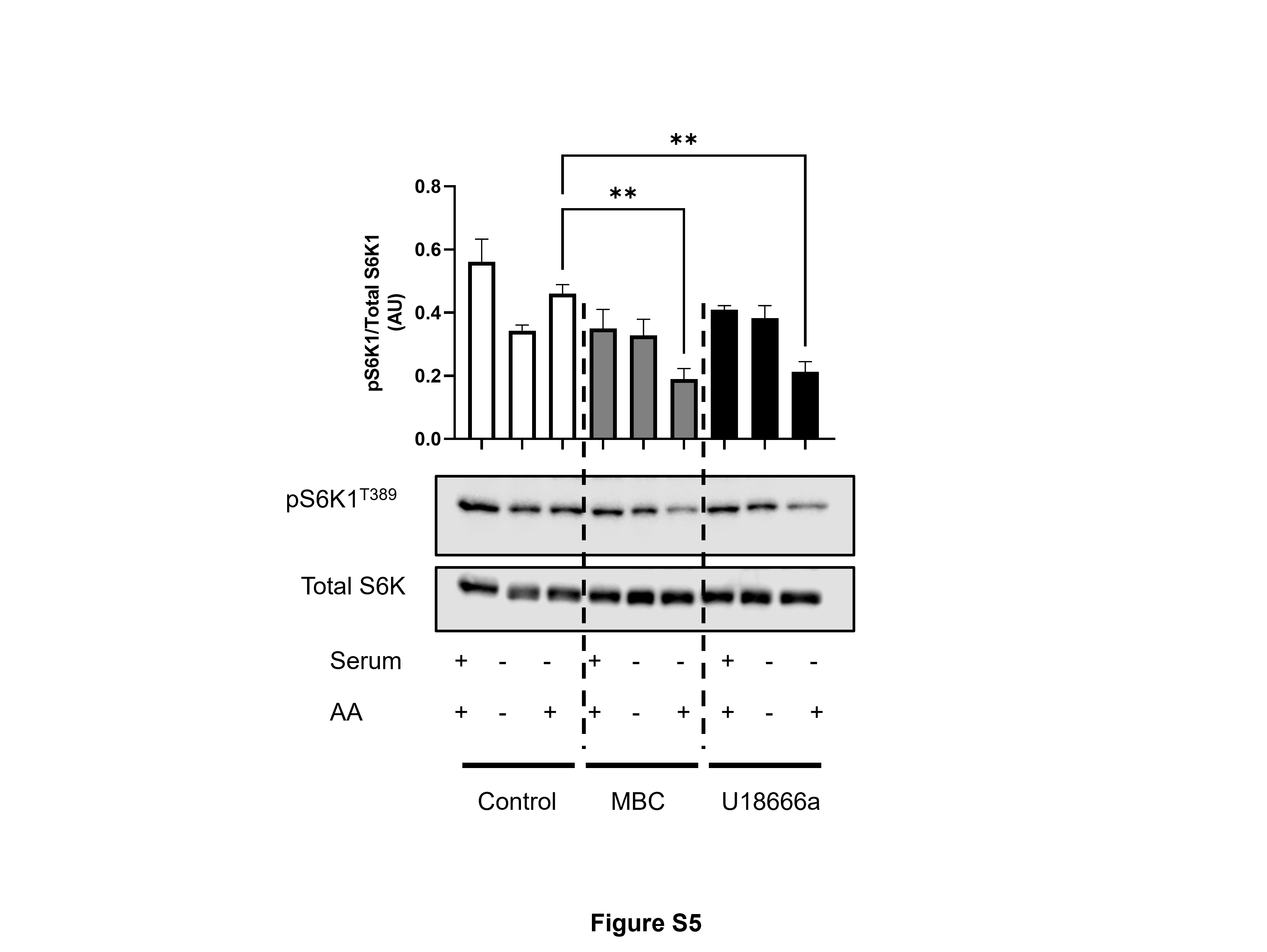

Supplement: Supplementary file 5 — Figure S5: Amino acid stimulated mTORC1 signalling is impaired when lysosomal cholesterol content is manipulated in LHCN‐M2 human muscle cells. Human LHCN‐M2 skeletal muscle cells (grown in DMEM/M199 medium (4:1) supplemented with penicillin streptomycin (100 μg/ml), FBS 15% (v/v) HEPES (20 mM), Zinc sulphate (30 ng/ml), vitamin B12 (1.4 μg/ml), dexamethasone (55 ng/ml), hepatocyte growth factor, recombinant human (2.5 ng/ml), and basic FGF (10 ng/ml)) were cholesterol depleted using 5 mM methyl‐β‐cyclodextrin for 2 h or pre‐treated with U18666a at 2.5 μg/mL for 16 h prior to serum and amino acid starvation using EBSS for 2 h. Muscle cells were subsequently refed with AA for 20 min. [file JCSM-14-2310-s005.tif]

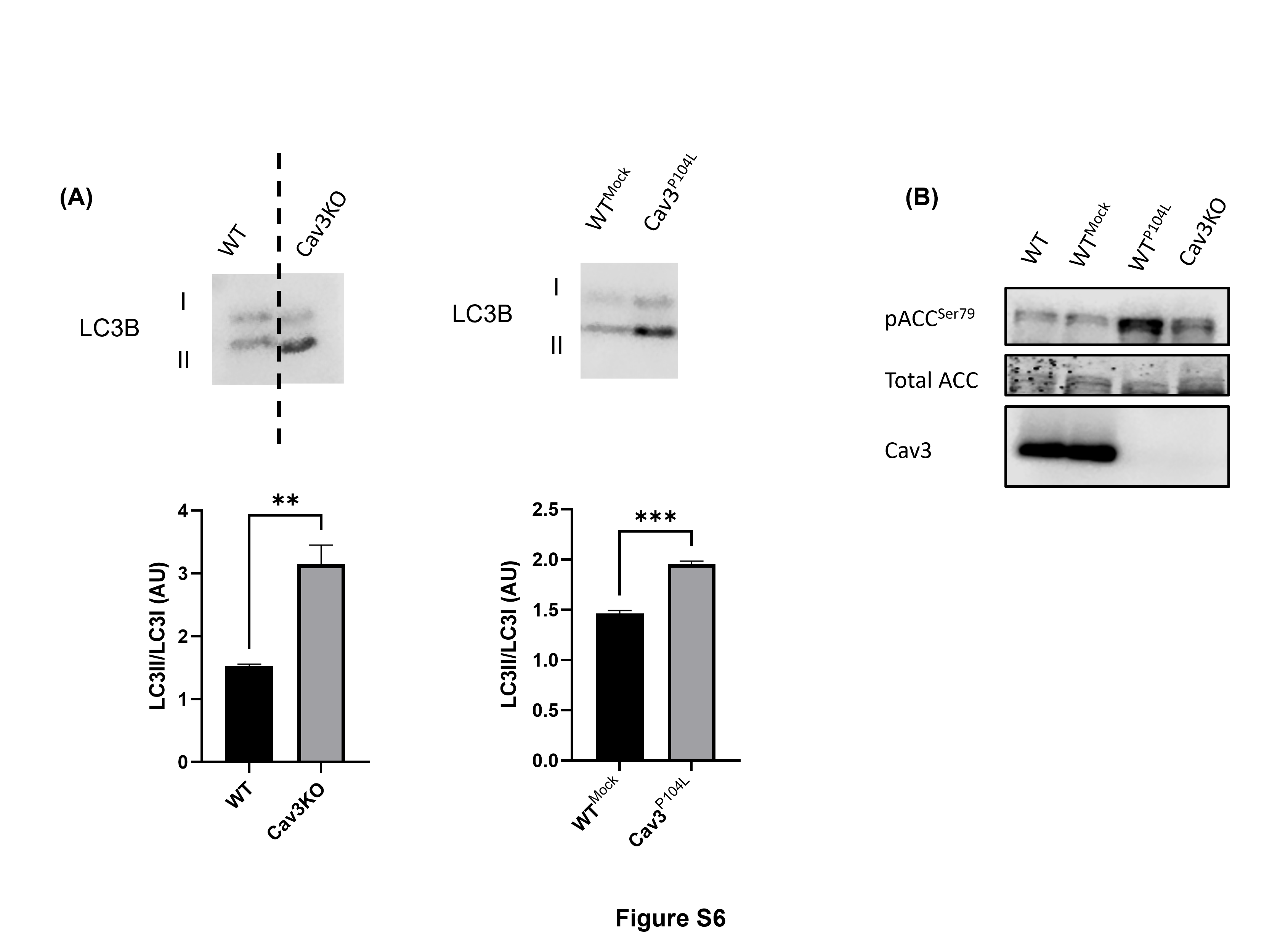

Supplement: Supplementary file 6 — Figure S6: Assessment of microtubule‐associated protein light chain 3 (LC3) lipidation and AMPK activation LC3 lipidation (LC3II) was assessed and compared to relative non‐lipidated LC3 (LC3I) and used as a readout for autophagic flux in WT or WTmock muscle cells compared with Cav3KO or Cav3P104L expressing cells respectively. Phosphorylation of an AMPK substrate Acetly CoA Carboxylase (pACCSer79) was assessed in response to Cav3 depletion. [file JCSM-14-2310-s007.tif]

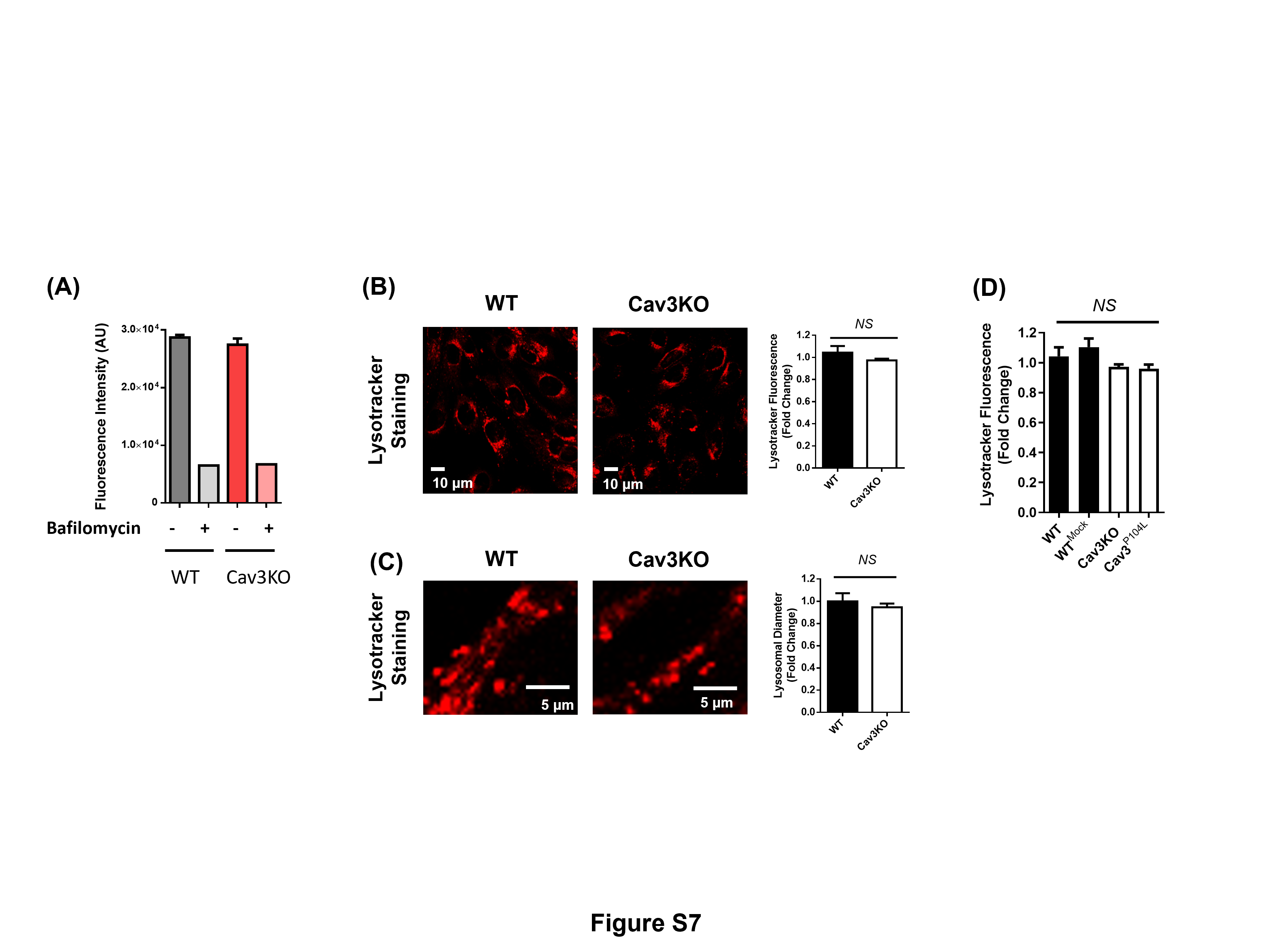

Supplement: Supplementary file 7 — Figure S7: Lysotracker staining in wild type L6 muscle cells or muscle cells deficient in Cav3. Wild type (WT) L6 myoblasts, Cav3KO L6 myoblasts, L6 myoblasts in which an empty vector or the Cav3P104L mutant was expressed were used for FACS analysis, microscopy or microplate readings. Lysosomal acidification was determined using FACS analysis of WT and Cav3KO myoblasts by assessing Lysotracker Deepred staining before or after pre‐treatment of cells with 200 nM of Bafilomycin. Following treatment muscle cells were fixed with 4% (w/v) PFA prior to analysis at excitation/emission wavelengths of 647/668 nm and analysis of mean geometric fluorescence intensity (A) or imaging using confocal microscopy (B/C). Volocity software was used to determine the mean fluorescence intensity from Lysotracker stained cells within confocal images (B) or lysosomal diameter (C). Alternatively, a plate reader was used to determine fluorescence intensity of Lysotracker stained cells (D). Bar graphs represent triplicate mean values ± SEM where NS indicates no significant difference. [file JCSM-14-2310-s008.tif]

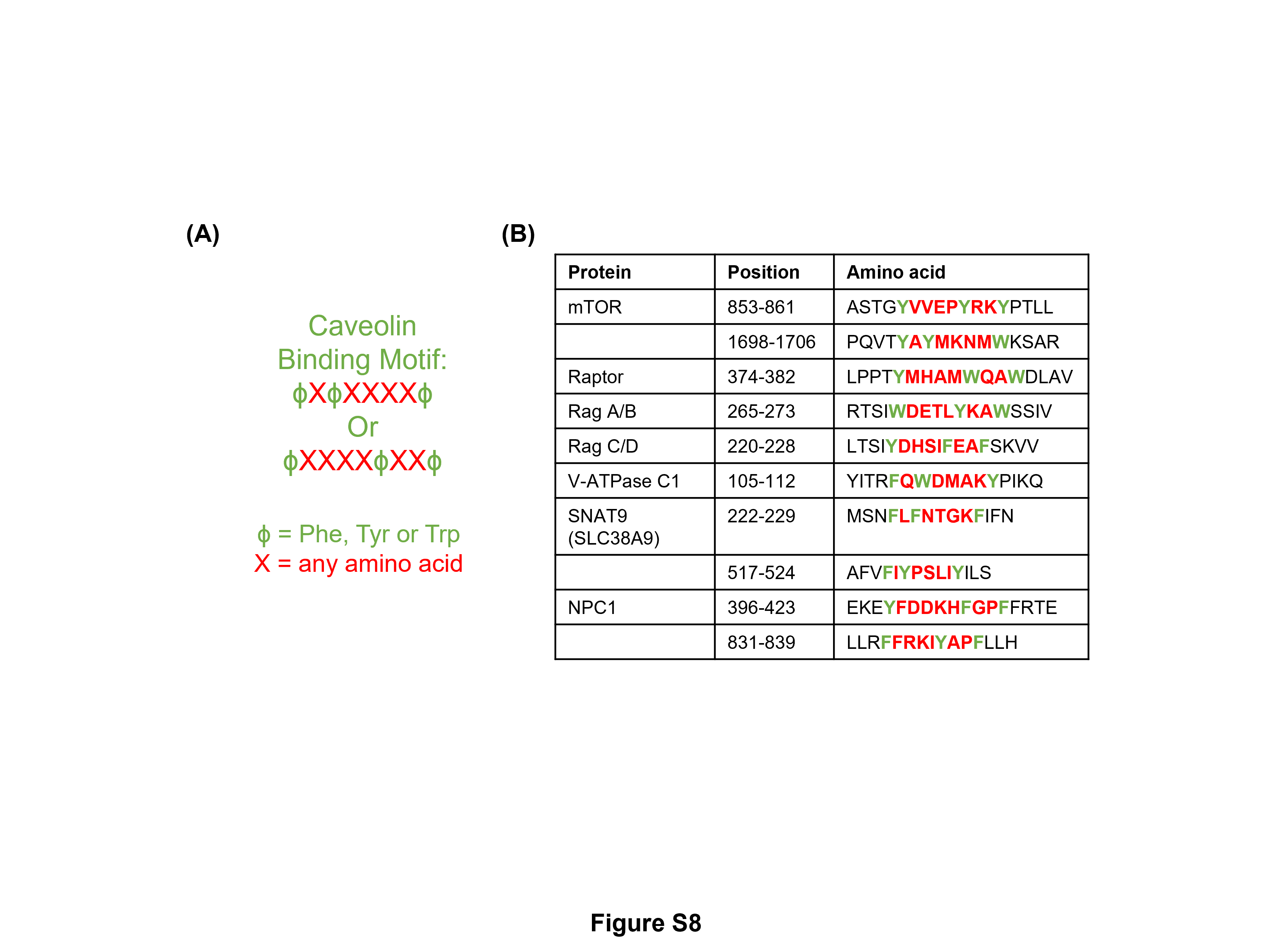

Supplement: Supplementary file 8 — Figure S8: In Silico identification of caveolin binding motifs (CBM) within proteins involved in lysosomal cholesterol homeostasis and mTORC1 activation. Caveolin binding motifs (CBM) (A) were identified by in silico analysis in a number of proteins implicated in the activation of mTORC1 (B). [file JCSM-14-2310-s003.tif]
